# Clathrin-mediated endocytosis facilitates the internalization of *Magnaporthe oryzae* effectors into rice cells

**DOI:** 10.1093/plcell/koad094

**Published:** 2023-03-28

**Authors:** Ely Oliveira-Garcia, Tej Man Tamang, Jungeun Park, Melinda Dalby, Magdalena Martin-Urdiroz, Clara Rodriguez Herrero, An Hong Vu, Sunghun Park, Nicholas J Talbot, Barbara Valent

**Affiliations:** Department of Plant Pathology, Kansas State University, Manhattan, KS 66506, USA; Department of Plant Pathology and Crop Physiology, Louisiana State University Agricultural Center, Baton Rouge, LA 70803, USA; Department of Plant Pathology, Kansas State University, Manhattan, KS 66506, USA; Department of Horticulture and Natural Resources, Kansas State University, Manhattan, KS 66506, USA; Department of Horticulture and Natural Resources, Kansas State University, Manhattan, KS 66506, USA; Department of Plant Pathology, Kansas State University, Manhattan, KS 66506, USA; School of Biosciences, University of Exeter, Exeter, EX4 4QD, UK; School of Biosciences, University of Exeter, Exeter, EX4 4QD, UK; The Sainsbury Laboratory, University of East Anglia, Norwich Research Park, Norwich NR4 7UH, UK; Department of Plant Pathology and Crop Physiology, Louisiana State University Agricultural Center, Baton Rouge, LA 70803, USA; Department of Horticulture and Natural Resources, Kansas State University, Manhattan, KS 66506, USA; The Sainsbury Laboratory, University of East Anglia, Norwich Research Park, Norwich NR4 7UH, UK; Department of Plant Pathology, Kansas State University, Manhattan, KS 66506, USA

## Abstract

Fungi and oomycetes deliver effectors into living plant cells to suppress defenses and control plant processes needed for infection. Little is known about the mechanism by which these pathogens translocate effector proteins across the plasma membrane into the plant cytoplasm. The blast fungus *Magnaporthe oryzae* secretes cytoplasmic effectors into a specialized biotrophic interfacial complex (BIC) before translocation. Here, we show that cytoplasmic effectors within BICs are packaged into punctate membranous effector compartments that are occasionally observed in the host cytoplasm. Live cell imaging with fluorescently labeled proteins in rice (*Oryza sativa*) showed that these effector puncta colocalize with the plant plasma membrane and with CLATHRIN LIGHT CHAIN 1, a component of clathrin-mediated endocytosis (CME). Inhibiting CME using virus-induced gene silencing and chemical treatments resulted in cytoplasmic effectors in swollen BICs lacking effector puncta. By contrast, fluorescent marker colocalization, gene silencing, and chemical inhibitor studies failed to support a major role for clathrin-independent endocytosis in effector translocation. Effector localization patterns indicated that cytoplasmic effector translocation occurs underneath appressoria before invasive hyphal growth. Taken together, this study provides evidence that cytoplasmic effector translocation is mediated by CME in BICs and suggests a role for *M. oryzae* effectors in coopting plant endocytosis.

## Teaser

Live cell imaging, virus-induced gene silencing, and pharmacological studies provide evidence that clathrin-mediated endocytosis facilitates translocation of *Magnaporthe* effectors inside rice cells.

**IN A NUTSHELL**

**Background:** To cause disease in plants, fungal pathogens deliver effector proteins directly into plant cells. Inside the host, effectors suppress the plant immune system and enable pathogens to rapidly invade and proliferate within plant tissue. How effector proteins enter plant cells, however, is not understood. During plant infection, the devastating rice blast fungus forms a specialized interfacial region known as the biotrophic interfacial complex (BIC), which is necessary for effector delivery into plant cells. We set out to explore how the BIC fulfils this role and how effectors enter plant cells.

**Question:** How does the blast fungus deliver effector proteins across the plasma membrane into living rice cells? Does the effector delivery system involve endocytosis by plant cells?

**Findings:** Live cell imaging of the blast fungus growing in rice cells provides evidence that effector proteins are packaged in dynamic vesicle-like membranous effector compartments (MECs) at the BIC. These MECs are bounded by the rice plasma membrane and CLATHRIN LIGHT CHAIN 1. Inhibition of endocytosis by chemical treatment or silencing rice genes involved in clathrin-mediated endocytosis prevents MEC formation and pathogenicity. An effector, Bas83, appears to play a role in recruiting fragments of plant membrane for endocytosis at the BIC. Taken together, our results provide evidence that clathrin-mediated endocytosis is necessary for effector translocation into plant cells.

**Next steps:** We would like to know how the blast fungus induces MEC formation; how effectors are released from MECs into the rice cytoplasm; and if, and if so how, Bas83 recruits plant membrane to promote MEC formation. It is important to identify key effectors involved in coopting host endocytosis to enable internalization of cytoplasmic effectors.

## Introduction

Many filamentous eukaryotic plant pathogens, such as fungi and oomycetes, cause plant disease by hijacking and feeding on living plant cells, and they deliver effectors into and around host cells to promote infection (Giraldo and Valent 2013; Lo Presti and Kahmann 2017). This includes the Ascomycete fungus *Magnaporthe oryzae* (synonym of *Pyricularia oryzae*), which threatens global food security by causing blast diseases on rice (*Oryza sativa*), on several millets, and most recently on wheat (*Triticum aestivum*) (Gladieux et al. 2018; Valent et al. 2020). *M. oryzae* executes a hemibiotrophic lifestyle initially involving biotrophic invasion of successive plant cells by specialized intracellular invasive hyphae (IH) that grow from cell to cell (Kankanala et al. 2007). Blast IH growing in living host cells are enclosed by extensions of the plant plasma membrane termed the extrainvasive hyphal membrane (EIHM). Colonized cells die as they fill with hyphae, and both the EIHM and host vacuolar membranes become disrupted. Meanwhile, the fungus moves on to colonize neighboring cells using the same invasion strategy (Kankanala et al. 2007; Mochizuki et al. 2015; Sakulkoo et al. 2018; Jones et al. 2021).

Live cell imaging of blast IH invading optically clear rice leaf sheath cells has defined a dimorphic hyphal switch that is essential for successful infection and is associated with the secretion and targeting of effectors—small, secreted proteins that control plant processes to promote infection. Specifically, cytoplasmic effectors, destined for translocation inside the host cell, are associated with the first 2 IH cells to grow in the plant cell lumen: the tubular primary invasive hypha that grows directly after penetration and the first pseudohyphal-like bulbous IH cell that results from hyphal differentiation (Kankanala et al. 2007; Khang et al. 2010). Effectors destined for delivery to the host cytoplasm are secreted by a specialized, brefeldin A (BFA)-insensitive, Golgi-independent secretion system (Giraldo et al. 2013).

After secretion, cytoplasmic effectors accumulate in an extended dome-shaped interfacial region of the EIHM matrix, the biotrophic interfacial complex (BIC). BICs first occur as “tip-BICs" at primary hyphal tips, and then as “side-BICs" that move beside the first bulbous IH cell (Khang et al. 2010). During live cell imaging, fluorescent cytoplasmic effectors are routinely detected in the cytoplasm and/or nuclei of invaded plant cells. Some cytoplasmic effectors are also detected in the cytoplasm of surrounding plant cells, apparently moving through plasmodesmata to prepare neighboring cells before invasion (Khang et al. 2010). By contrast, apoplastic effectors, such as the Biotrophy-associated secreted protein 4 (Bas4), the LysM protein 1 (Slp1), and Bas113, are secreted via conventional Golgi-dependent secretion and accumulate in the EIHM around the entire IH (Giraldo et al. 2013). The retention of apoplastic effectors such as Bas4 inside the EIHM (Mosquera et al. 2009; Khang et al. 2010), lack of separation of the EIHM from IH during plasmolysis (Kankanala et al. 2007), and exclusion of the endocytosis tracker dye FM4-64 from IH membranes inside the FM4-64 stained EIHM (Kankanala et al. 2007; Giraldo et al. 2013) all indicate that IH grow inside a sealed EIHM compartment during the biotrophic invasion of plant cells.

The mechanisms by which eukaryotic filamentous plant pathogens deliver effectors across the host plasma membrane into living host cells are poorly understood and may be evolutionarily conserved or unique for different host-pathogen interactions (Giraldo and Valent 2013; Petre and Kamoun 2014; Fawke et al. 2015; Lo Presti et al. 2015; Lo Presti and Kahmann 2017). The oomycete potato (*Solanum tuberosum*) pathogen *Phytophthora infestans* resembles *M. oryzae* in having a BFA-insensitive, Golgi-independent secretion system for cytoplasmic effectors (Giraldo et al. 2013; Van den Ackerveken 2017; Wang et al. 2017). Although cell entry motifs for fungal effectors have not been identified, oomycete effectors contain amino acid translocation motifs, with the conserved N-terminal motif Arg-X-Leu-Arg (RXLR, with X being any amino acid) being the most common. The RXLR motif in oomycete effectors was reported to mediate host cell uptake through endocytosis based on its binding to phosphatidylinositol-3-phosphate (PI3P) in the host membrane (Kale et al. 2010), although it was subsequently shown that the RXLR motif from the *P. infestans* effector AVR3a is cleaved off before secretion from the pathogen (Wawra et al. 2017). Therefore, the function of the RXLR motif in cell entry remains disputed (Petre and Kamoun 2014; Trusch et al. 2018).

Another amino acid motif, Tyr-Lys-Ala-Arg-Lys (YKARK, in the C-terminus), appears to be involved in translocation of the effector SpHtp3 from *Saprolegnia parasitica*, a serious oomycete pathogen of fish, inside fish cells (Trusch et al. 2018). Specifically, this effector appears to be internalized inside host cells via lipid raft-mediated endocytosis through binding to a gp96-like receptor, and another effector is involved in releasing SpHtp3 from vesicles into the fish cytoplasm. In an example from a fungal pathogen, a solvent-exposed Arg-Gly-Asp (RGD) vitronectin-like motif is required for the internalization of ToxA, a host-specific proteinaceous toxin secreted by the necrotrophic wheat pathogen *Pyrenophora tritici-repentis* (Manning and Ciuffetti 2005). In addition to uptake by plant endocytosis, effectors might enter plant cells through specialized translocon complexes in the plasma membrane. For example, the human malaria parasite *Plasmodium falciparum* is contained within a parasitophorous vacuole inside invaded red blood cells, and effectors are delivered across the membrane into the host cytoplasm by the Plasmodium translocon of exported proteins translocon (Elsworth et al. 2014). A protein complex comprising 5 unrelated fungal effectors and 2 fungal membrane proteins was recently implicated in effector translocation by the maize (*Zea mays*) smut fungus *Ustilago maydis* (Ludwig et al. 2021). However, the general question of how effectors are taken up into plant cells remains largely unresolved for eukaryotic plant pathogens.

Clathrin-mediated endocytosis (CME) is the major mechanism by which eukaryotic cells internalize extracellular or membrane-bound cargoes. This process is best understood in mammalian systems and yeast (Kaksonen and Roux 2018). CME in plants shows both conserved and evolutionarily unique mechanistic details (Qi et al. 2018; Reynolds et al. 2018; Dejonghe et al. 2019; Johnson et al. 2020; Narasimhan et al. 2020; Aniento et al. 2021). CME is known to play key roles in plant-microbe interactions (Dodds and Rathjen 2010; Beck et al. 2012; Fan et al. 2015). CME is required for immunity mediated by pattern recognition receptor (PRR) kinases, for instance, through the internalization of activated extracellular PRRs for degradation in the vacuole (Mbengue et al. 2016). Examples include the PRR PEP RECEPTOR 1/2 (PEPR1/2), which recognizes damage-associated endogenous plant peptides; the EF-TU receptor (EFR), which recognizes the pathogen-associated molecular pattern (PAMP) bacterial Elongation Factor TU; FLAGELLIN-SENSING 2 (FLS2), which recognizes the PAMP flagellin (flg-22) (Spallek et al. 2013; Mbengue et al. 2016); and the Cf-4 receptor-like protein, which recognizes the *Cladosporium fulvum* Avr4 avirulence effector (Postma et al. 2016).

Additionally, pathogens can control plant CME. In addition to its role in suppressing INF1-mediated cell death, the *P. infestans* host-translocated RXLR avirulence effector AVR3a associates with Dynamin-Related Protein 2 (a plant GTPase implicated in receptor-mediated endocytosis), suppresses flg22-triggered defense responses, and reduces FLS2 internalization (Chaparro-Garcia et al. 2015). Therefore, AVR3a interacts with a membrane-remodeling complex involved in immune receptor-mediated endocytosis. Also, in host cells containing haustoria from the oomycete *P. infestans*, the late endosomal pathway is rerouted from the vacuole to the extrahaustorial membrane (EHM) at the haustorial interface (Bozkurt et al. 2015). Multiple effectors from this pathogen are implicated in rerouting the host membrane trafficking system and autophagy machinery for the benefit of the invading pathogen (Bozkurt et al. 2015; Dagdas et al. 2018; Petre et al. 2021).

Effector translocation in the *M. oryzae*/rice pathosystem can be assayed directly by live cell imaging of fluorescently labeled effector proteins inside host cells after secretion by IH invading rice leaf sheath cells (Khang et al. 2010). Blast cytoplasmic effectors apparently do not contain a translocation motif as reported for oomycete effectors. Instead, preferential accumulation in the BIC is the major predictor for translocation into the rice cell. Nishizawa and colleagues reported that a cytoplasmic effector occurs in puncta in an outer BIC region (Mochizuki et al. 2015; Nishimura et al. 2016; Nishizawa et al. 2016). Here, we have used live cell fluorescence imaging to characterize the nature and dynamics of vesicle-like membranous effector compartments (MECs) in BICs and the surrounding host cytoplasm. The membranous compartments containing cytoplasmic effectors at BICs colocalized with fluorescently labeled CLATHRIN LIGHT CHAIN 1. We performed functional analyses of plant endocytosis components using virus-induced gene silencing (VIGS) and pharmacological approaches. Together, our data indicate that blast effectors are internalized through host CME in the outer BIC layers, followed by escape from the MEC into the rice cytoplasm.

## Results

### Cytoplasmic effectors localize in MECs in and around BICs

**Figure 1.**
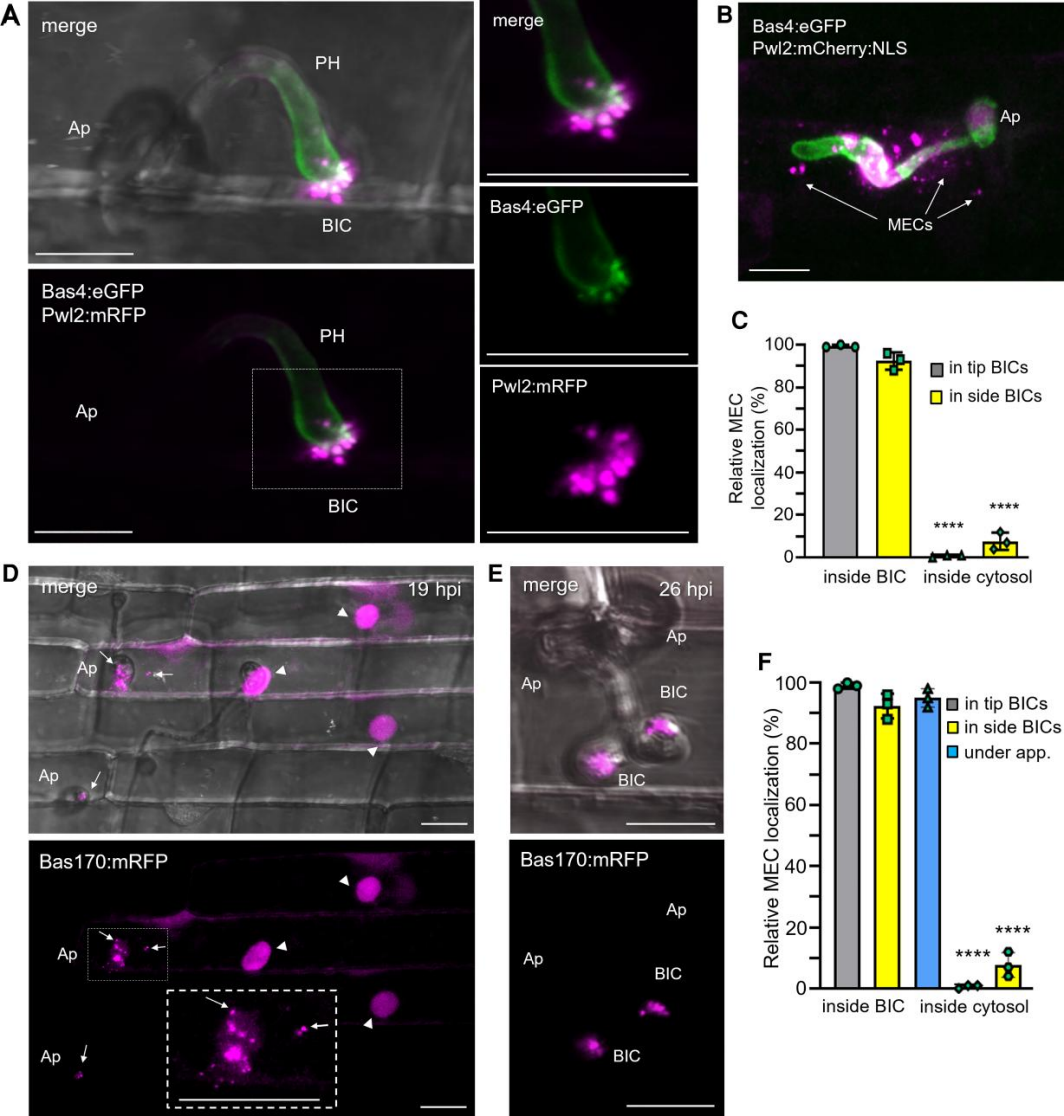
Cytoplasmic effectors are organized in punctate MECs coinciding with translocation inside the host cell. **A)** Cytoplasmic effector-labeled MECs in a BIC at the tip of a primary hypha (PH) of strain KV217 expressing Pwl2:mRFP (magenta) and Bas4:eGFP (green) while invading a rice sheath epidermal cell at 17 h post inoculation (hpi). The insets on the right (corresponding to the white box, lower left) show merged and individual fluorescence channels. The appressorium (Ap) and the first section of the PH are out of focus. **B)** A BIC above a first bulbous IH cell of strain KV168 expressing Pwl2:mCherry:NLS (magenta) and Bas4:eGFP at 24 hpi. MECs are visible in the host cytoplasm at a distance from the BIC (arrows). Bas4:eGFP outlining of the IH shows that the surrounding EIHM remains intact. **C)** Quantification of the relative localization of cytoplasmic effector MECs carrying Pwl2:mRFP (strain KV217, panel **A**). Ninety-eight MECs were counted for BICs at the tips of PH (tip-BICs) and for BICs beside the first bulbous IH cell (side-BICs). Bar charts are based on means of all data from 3 biological reps (data points show means of the individual reps); error bars indicate Sd. ****P < 0.0001. **D)** Bas170:mRFP (strain KV224) accumulates in MECs (white arrows) under Ap and in rice nuclei, indicating translocation (arrowheads), even before PH are visible. An enlarged view of MECs near a penetration site is shown in the inset on the right. **E)** Bas170:mRFP also localizes in MECs at later invasion stages, shown in 2-side BICs resulting from penetration by adjacent Ap. Experimental details same as in D. **F)** Quantification of the relative localizations of Bas170:mRFP-labeled MECs, assessed using strain KV224 (**D** and **E**). 100 MECs counted for each development stage. Bar charts same as described in (**C**). **A**, **B**, **D**, and **E**) All images are projections of confocal optical sections. Single or dual fluorescence channels shown are listed, with merge images on the left also including bright field. Scale bars = 10 µm.

We previously described BICs as interfacial structures containing fluorescently labeled apoplastic effectors in an inner base layer and fluorescent cytoplasmic effectors in an outer dome-shaped region (Khang et al. 2010). Here, using laser confocal imaging, we resolved the BIC outer dome in rice cells as a concentration of puncta identified by cytoplasmic effector fluorescence (Fig. 1). Based on the totality of evidence, we refer to these cytoplasmic effector puncta as MECs, as illustrated by an image of a BIC at the tip of a primary hypha expressing the apoplastic effector Bas4 fused to enhanced green fluorescent protein (Bas4:eGFP) and cytoplasmic effector Pwl2 (Pathogenicity towards weeping lovegrass 2) fused to monomeric red fluorescent protein (Pwl2:mRFP) (Fig. 1A). In this image, the outline of fluorescence of apoplastic Bas4:eGFP around the primary hypha indicates that the EIHM surrounding the growing hypha is intact. By contrast, Pwl2:mRFP occurs as distinct MEC puncta in the outer BIC region. MEC formation is independent of the cytoplasmic effector or fluorescent protein used (Supplemental Fig. S1). MECs are also observed in BICs that are left behind beside the initial bulbous IH cells after hyphal differentiation (Fig. 1B). Whereas the vast majority of MECs associated with primary hyphae were located in the BIC, MECs associated with bulbous IH cells were found in BICs as well as outside the BIC, which we define as the location in the host cytoplasm at least 7 µm from the fungal cell wall at the BIC (Fig. 1C). Uniform outlining of the entire IH by Bas4:eGFP (Fig. 1B) again confirmed that the EIHM was intact and that MECs had not spilled into the invaded cell due to rupture of the EIHM.

MEC organization of cytoplasmic effectors corresponds with the period of active translocation of effectors into the rice cytoplasm. For example, a larger view of the infection site in Fig. 1B shows that the cytoplasmic effector PWL2:mCherry:NLS (Pwl2 fused to mCherry fluorescent protein and a C-terminal nuclear localization signal, NLS) has been translocated into the host cell (Supplemental Fig. S2). Adding a NLS to an effector fusion protein (Khang et al. 2010) facilitates observation of the translocated fluorescent signal in the nuclei of both invaded and neighboring cells. MECs in another BIC (Supplemental Movie S1) were observed together with effector fluorescence in the cytoplasm of the invaded host cell, including in a cytoplasmic connection linking the BIC to plant cytoplasm at the cell periphery.

A previously uncharacterized cytoplasmic effector, Bas170 (Supplemental Fig. S3), exhibited a different localization pattern in the initially invaded rice cells. Unlike other cytoplasmic effectors including Pwl2:mRFP, fluorescent Bas170 fusion proteins localized to punctate MECs in the rice cytoplasm short distances underneath appressoria before primary hyphae became visible (Fig. 1, D and F). At the same time, Bas170:mRFP fusion protein naturally accumulated in rice nuclei, suggesting rapid effector translocation from appressoria even before the growth of primary IH (Fig. 1D). Bas170:mRFP also labeled MECs in BICs following the growth of IH in the cell (Fig. 1, E and F). We conclude that both the timing and properties of MECs are consistent with the notion that these structures play a role in blast effector translocation.

### MECs in BICs are dynamic

**Figure 2.**
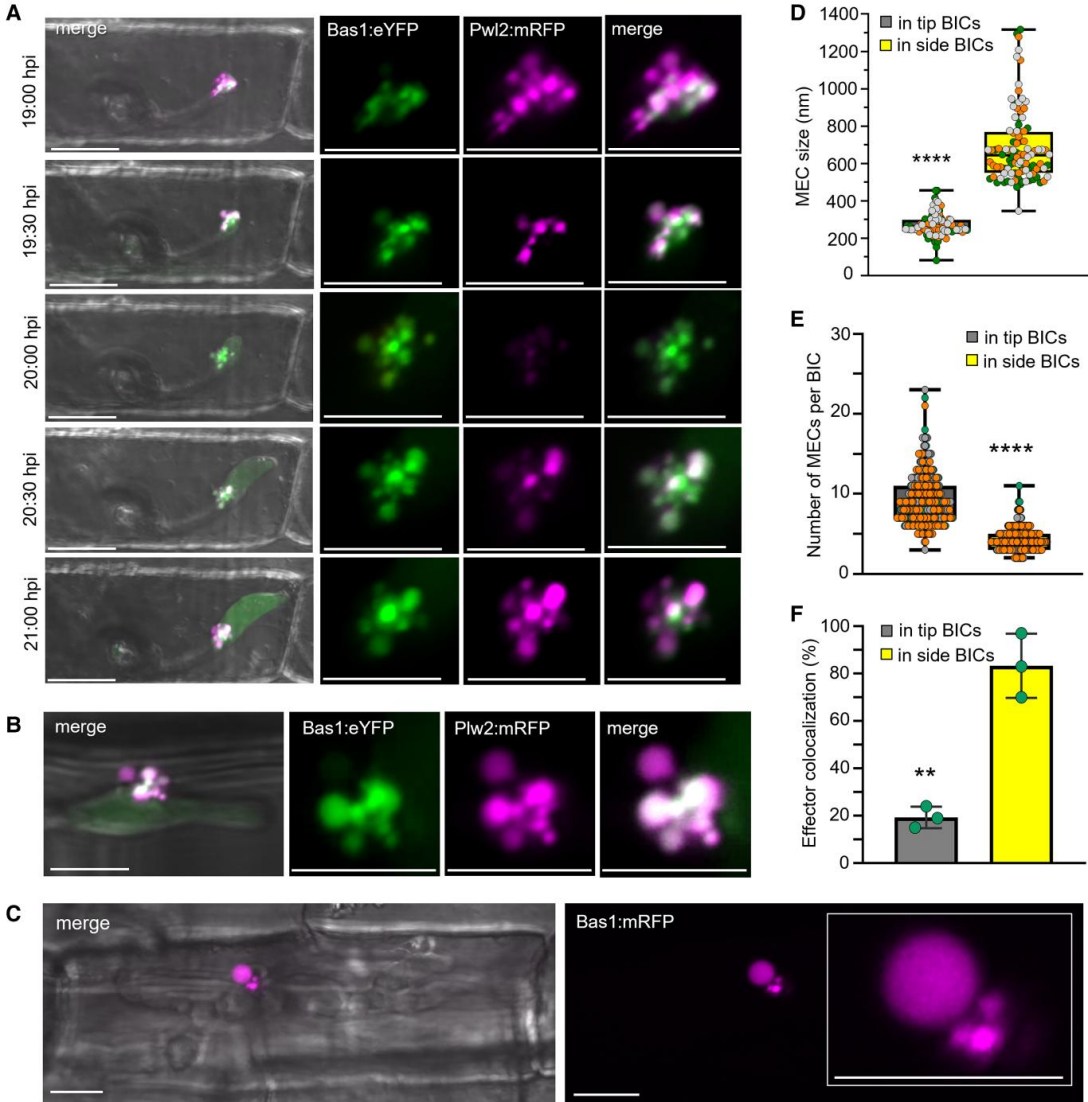
MEC dynamics during invasive hyphal differentiation and growth. **A)** Cytoplasmic effectors Pwl2:mRFP and Bas1:eYFP (strain KV211) were observed as BICs move from primary hyphal tips (top panel) to beside the first bulbous IH cell (bottom panel), imaged every 30 min. Left to right: Merged bright field, eYFP (green), and mRFP (magenta) fluorescences, with enlarged views of eYFP alone, mRFP alone, and merged eYFP and mRFP. Images are projections of confocal optical sections. **B)** Cytoplasmic effectors Pwl2:mRFP and Bas1:eYFP (strain KV211) colocalize in larger MECs in a later stage BIC after further growth of bulbous IH (24 hpi). Experimental details are the same as in A. **C)** A rare extremely large MEC (diameter 3.9 µm) in a late stage BIC after branching bulbous IH growth by KV170 expressing Bas1:mRFP (magenta) at 30 hpi. Shown left to right are merged bright field and mRFP fluorescence; and mRFP alone (enlarged view in the inset). **D** and **E**) Box-and-whisker plots with individual data points comparing MEC sizes, as measured by fluorescence, (**D**) and numbers (**E**) in tip- and side-BICs formed by strain KV211. Data points with different colors represent different biological replicates. **F**) Bar chart showing quantification of colocalization of Bas1:eYFP and Pwl2:mRFP (strain KV211) in MECs in tip- and side-BICs. Bar charts are based on means of all data from 3 biological replicates with the data points showing the means of the individual reps; error bars indicate Sd. **A** to **C**) Scale bars = 10 m. **D** to **F**) **P = 0.0015; ****P < 0.0001. One hundred twelve MECs assessed for tip- and side-BICs in (**D**). One hundred BICs observed per replicate in (**E** and **F**).

We previously used Fluorescence Recovery after Photobleaching to show that cytoplasmic effectors are continuously secreted into BICs when tubular primary hyphae differentiate into the first bulbous IH cells (Giraldo et al. 2013; Khang et al. 2010). Here, to explore MEC dynamics over time, we imaged effector fluorescence during this hyphal morphogenetic switch using a pathogen strain coexpressing 2 cytoplasmic effectors, Bas1:eYFP (Bas1 fused to enhanced yellow fluorescent protein) and Pwl2:mRFP (Fig. 2). MEC content varied over a 2-hour period as the BIC at a primary hyphal tip was displaced beside the differentiating bulbous IH cell (Fig. 2A). In BICs at primary hyphal tips, fluorescence from the 2 effectors often appeared in different MECs. Initially, Pwl2:mRFP occurred in MECs that reached the outer BIC layers compared to Bas1:eYFP. During hyphal differentiation, Pwl2:mRFP appeared to be less abundant relative to Bas1:eYFP and then recovered again after further growth of bulbous IH (Fig. 2A). Both the sizes and numbers of MECs within BICs varied through time (Fig. 2B to F). Specifically, early stage BICs at primary hyphal tips contained a larger number of smaller MECs (Fig. 2, D and E). Whereas MECs in hyphal tip-BICs had a median diameter of 249 nm, MECs in later stage BICs beside the first bulbous IH cell had a median diameter of 647 nm (ranging from ∼500 nm to >1000 nm) as subsequent bulbous IH cells grew to fill the invaded host cell (Fig. 2, C and D). Larger MECs beside mature bulbous IH often showed colocalization of fluorescence from different effectors compared to those in tip-BICs (Fig. 2, A and B and F). The dynamic nature of MECs is consistent with their possible role in releasing effectors into the host cytoplasm.

### MECs colocalize with the plant plasma membrane

**Figure 3.**
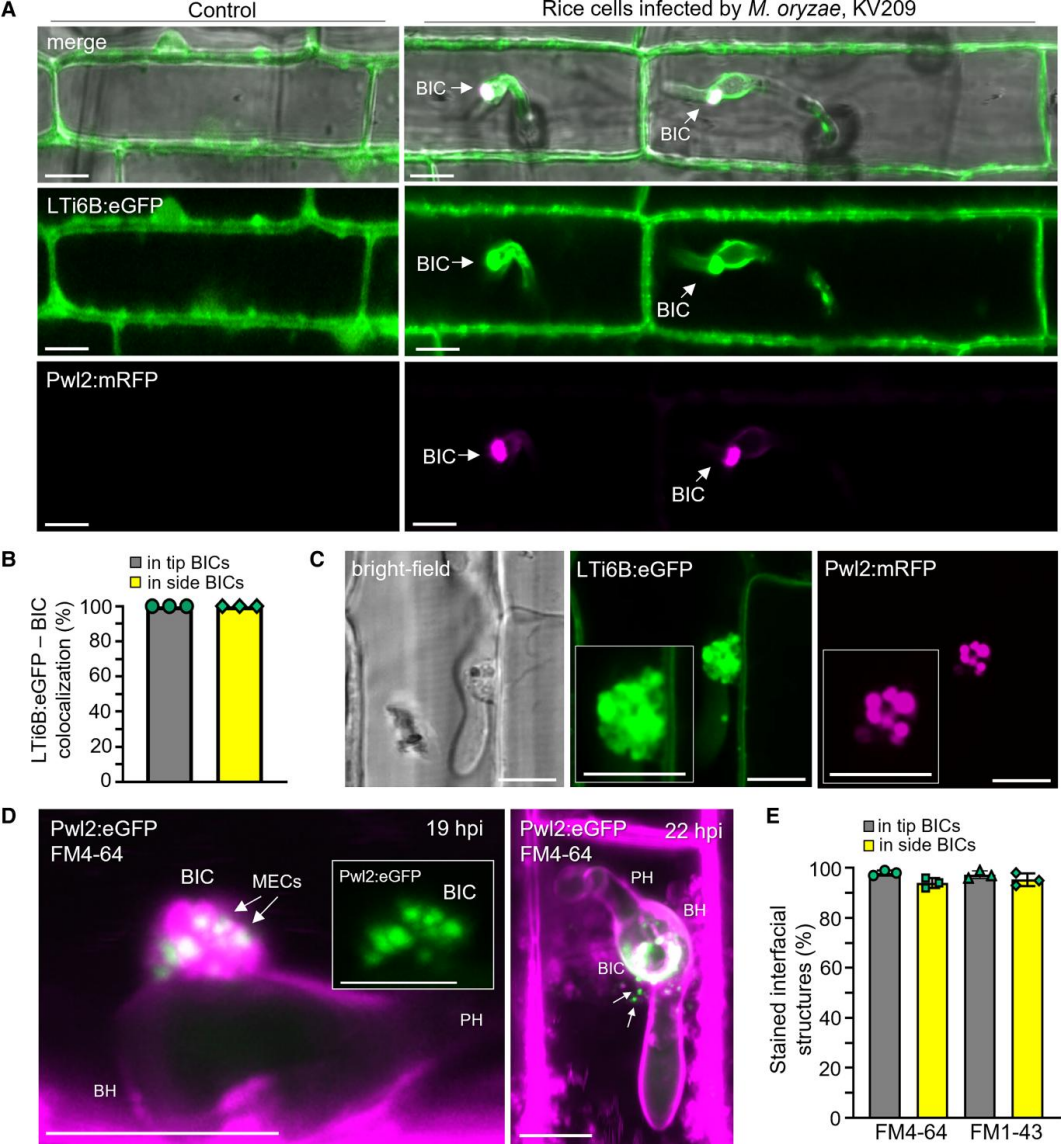
MECs colocalize with the plant plasma membrane. **A)** Rice expressing plasma membrane marker LTi6B:eGFP in noninfected cells (left) and in cells infected by strain KV209 (right) expressing Pwl2:mRFP (magenta). Colocalization is illustrated in independent side-BICs in adjacent rice cells. **B)** Quantification of the colocalization of LTi6B:eGFP with BICs. Bar charts are based on means of all data from 3 biological replicates and data points show the means of the individual reps. Three times 90 infection sites were observed. **C)** LTi6B:eGFP outlines individual MECs in a BIC identified by Pwl2:mRFP (strain KV209, 22 hpi). Shown are individual brightfield, eGFP, and mRFP images with enlarged views in insets. **D)** MECs in 2 BICs formed by strain KV176 (expressing Pwl2:eGFP) colocalize with FM4-64-stained plant membranes (magenta). On the left, MECs in a side-BIC at 19 hpi are immersed in FM4-64 (shown as merged eGFP and FM4-64 with eGFP alone in the inset). On the right, a top-down view of a different BIC at 22 hpi. Arrows indicate MECs near the BIC. Lack of FM4-64 staining of fungal septa and vacuolar membrane indicates intact EIHM surrounds each IH. **E)** Quantification of BICs stained by the endocytosis tracer dyes FM4-64 or FM1-43. Bar charts are based on means of all data from 3 biological replicates (data points show the means of the individual reps); error bars indicate standard deviation; 3 times 90 infection sites observed per treatment. **A** to **D**) Scale bars = 10 µm.

We confirmed our previous finding (Giraldo et al. 2013) that BICs are enriched in the plant plasma membrane by infecting transgenic rice expressing the plasma membrane marker LOW-TEMPERATURE INDUCED PROTEIN 6B fused to green fluorescent protein (LTi6B:GFP) (Fig. 3, A and B). We then used these same rice lines to observe the colocalization of the plant plasma membrane with individual MECs (Fig. 3C). To further confirm that MECs are associated with the plant membrane, we evaluated BIC membrane composition by staining with the endocytosis tracker dyes FM4-64 and FM1-43 (Fig. 3, D and E). These amphiphilic styryl dyes insert in plant and fungal plasma membranes where they diffuse laterally in these membranes and are internalized into cells via endocytosis (Atkinson et al. 2002; Bolte et al. 2004). For healthy IH growing in rice cells, FM4-64 heavily stains the EIHM surrounding IH but is excluded from IH membranes inside the sealed EIHM compartment. This is clearly visualized by a lack of lateral diffusion of the dye into IH septal membranes and a lack of accumulation in IH vacuolar membranes (Kankanala et al. 2007; Giraldo et al. 2013; Jones et al. 2021). Therefore, the exclusion of FM4-64 or FM1-43 staining from internal IH membranes served as a control for EIHM intactness at observed infection sites (Fig. 3D and Supplemental Movie S2). Quantification of FM4-64 and FM1-43 accumulation in both tip- and side-BICs (Fig. 3E) confirmed that MECs were immersed in the region of intense dye staining (Fig. 3D; Supplemental Movie S2). MECs separated from the BIC by at least 7 µm were infrequently observed (Fig. 3D, right; Supplemental Movie S2). Apparent MEC bursting was observed, which suggests that they are unstable, presumably releasing the effector contents into the host cytoplasm. Taken together, the results of labeling the rice plasma membrane with LTi6B:GFP fusion protein and endocytosis tracer dyes are consistent with the notion that BIC-associated MECs form via plant endocytosis.

### Bas83 labels an additional rice membrane compartment associated with BICs

**Figure 4.**
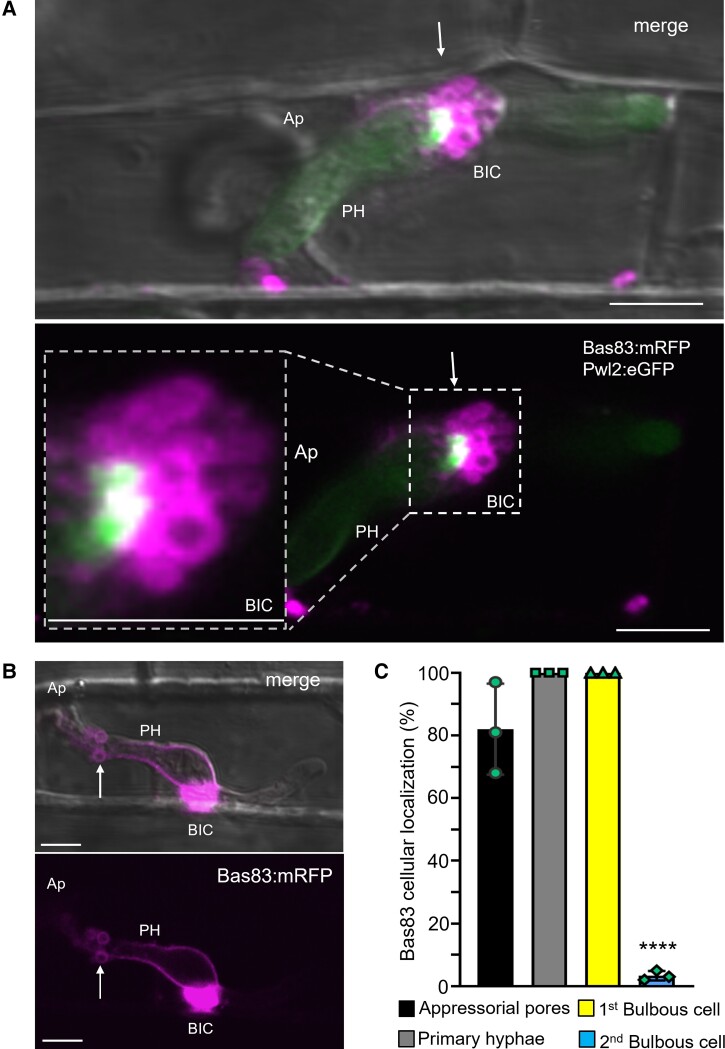
Cytoplasmic effector Bas83 labels additional membranous compartments surrounding BICs and BIC-associated cells. **A)** Bas83:mRFP (magenta) from strain KV222 expressing Bas83:mRFP and Pwl2:eGFP identifies membranous compartments that surround the BIC (white arrow) and lack fluorescence from the cytoplasmic effector (green). An enlarged view of this side-BiC is shown in the inset. **B)** Bas83:mRFP (strain KV220) labels the EIHM around the PH and membrane compartments nearby (white arrow), in addition to the BIC region. **C)** Quantification of the cellular localization of Bas83:mRFP during biotrophic invasion (strain KV220). Bar charts are based on means of all data from 3 biological replicates (data points show the means of the individual reps); error bars indicate standard deviation. ****P < 0.0001; 100 infection sites observed per experiment. **A** and **B**) Top images show bright field merged with dual or single fluorescence below. Scale bars = 10 µm.

We previously identified the effector gene *BAS83*, which is upregulated 36-fold during biotrophic invasion and is unique to *M. oryzae* (Mosquera et al. 2009). Here we show that fluorescent Bas83:mRFP fusion proteins had a unique localization pattern after translocation into invaded rice cells. Specifically, Bas83 was associated with membranous compartments concentrated around BICs and BIC-associated hyphal cells (Fig. 4, A and B; Supplemental Fig. S4). Bas83:mRFP also labeled the EIHM surrounding BIC-associated cells (Fig. 4B), suggesting potential fusion with the EIHM. Bas83:mRFP-labeled membranous compartments did not contain cytoplasmic effector fluorescence, indicating they are distinct from MECs (Fig. 4A). Concentrated Bas83:mRFP signals were also observed under appressoria (Fig. 4C; Supplemental Fig. S4). Bas83:mRFP failed to label membranes surrounding bulbous IH cells that were not associated with BICs (see 2nd bulbous IH, Fig. 4C). We hypothesize that Bas83 is involved in recruiting the plant membrane to support rapid membrane turnover in BICs. However, repeated attempts in 2 of our laboratories (264 purified transformants, 4 methods and 5 experiments; Kansas State University and University of Exeter) failed to produce knockout mutants for further functional analysis (Supplemental Fig. S5).

### BICs contain plant actin

**Figure 5.**
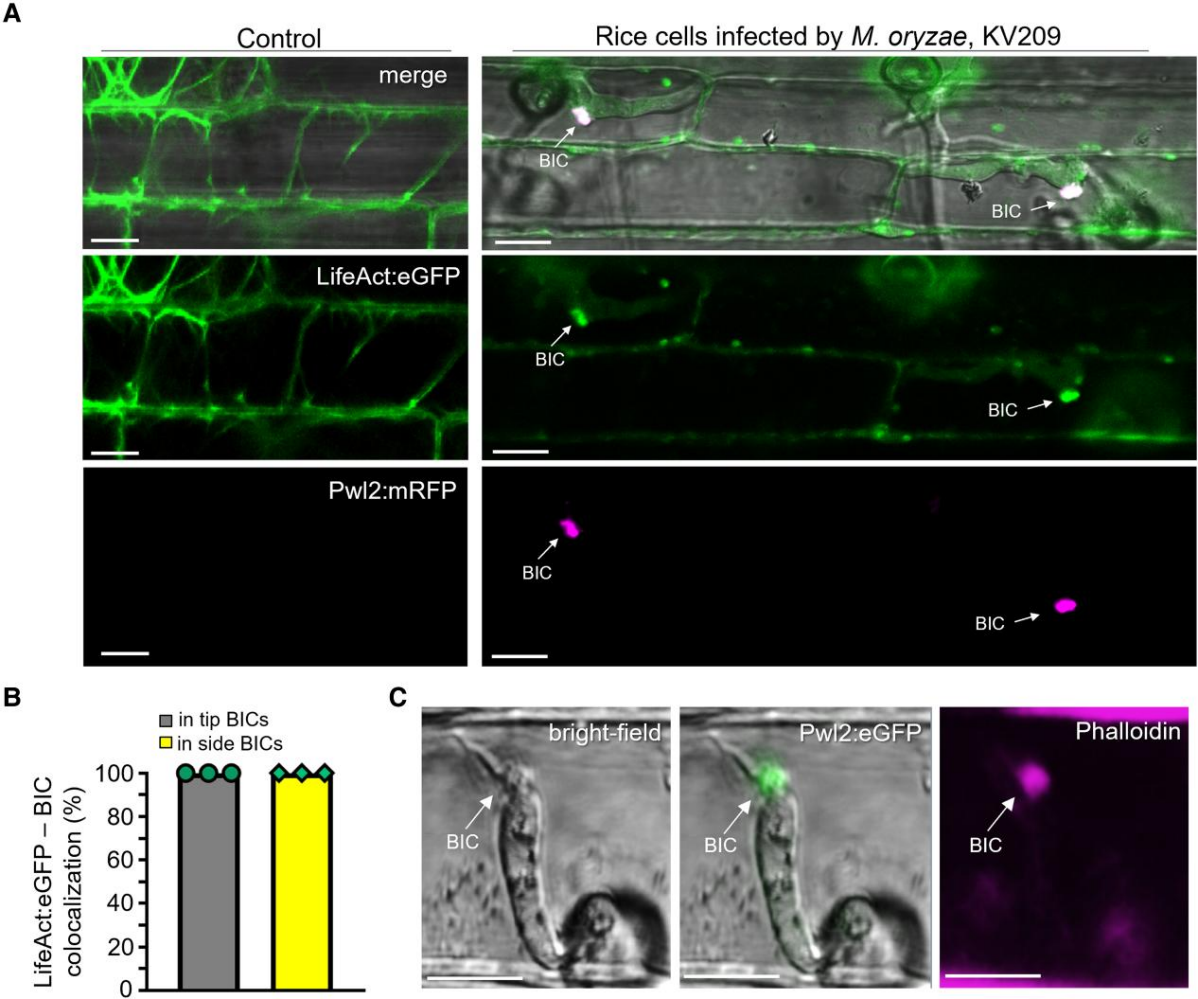
BICs are rich in plant actin. **A)** Imaging with a transgenic rice line expressing LifeAct:eGFP in noninfected cells (left) and in cells infected by strain KV209 expressing Pwl2:mRFP (right) shows actin concentrated in BIC regions. **B)** Bar charts quantifying colocalization of LifeAct:eGFP with tip- and side-BICs are based on means of combined data from 3 biological replicates (data points show means of the individual reps); error bars indicate standard deviation; 3 times 100 infection sites observed. **C)** Rhodamine phalloidin conjugate-stain for actin (magneta) colocalizes with Pwl2:eGFP (green) in rice cells infected by strain KV176, confirming the actin-rich structure of BICs. Scale bars = 10 µm.

Unlike mammals and yeast, actin is not required for the internalization of clathrin-coated vesicles (CCVs) in plants (Qi et al. 2018; Johnson et al. 2020; Narasimhan et al. 2020). However, actin filaments are likely to play a role in the short-distance movement of internalized vesicles (Schuh 2011; Ruan et al. 2021; Narasimham et al. 2020). Therefore, we localized plant actin during biotrophic invasion using rice lines expressing the actin marker LifeAct:eGFP. Infection by *M. oryzae* strains expressing the fluorescent effector Pwl2:mRFP showed localization of LifeAct:eGFP fluorescence at BICs (Fig. 5A). Actin was abundant in all imaged tip- and side-BICs (Fig. 5B). Likewise, colocalization of fluorescent Phalloidin conjugates and fluorescently labeled effector confirmed that BICs are rich in plant actin (Fig. 5C). We hypothesize that actin filaments are involved in the transport of MECs in the rice cytoplasm.

### MECs colocalize with fluorescent CLATHRIN LIGHT CHAIN

**Figure 6.**
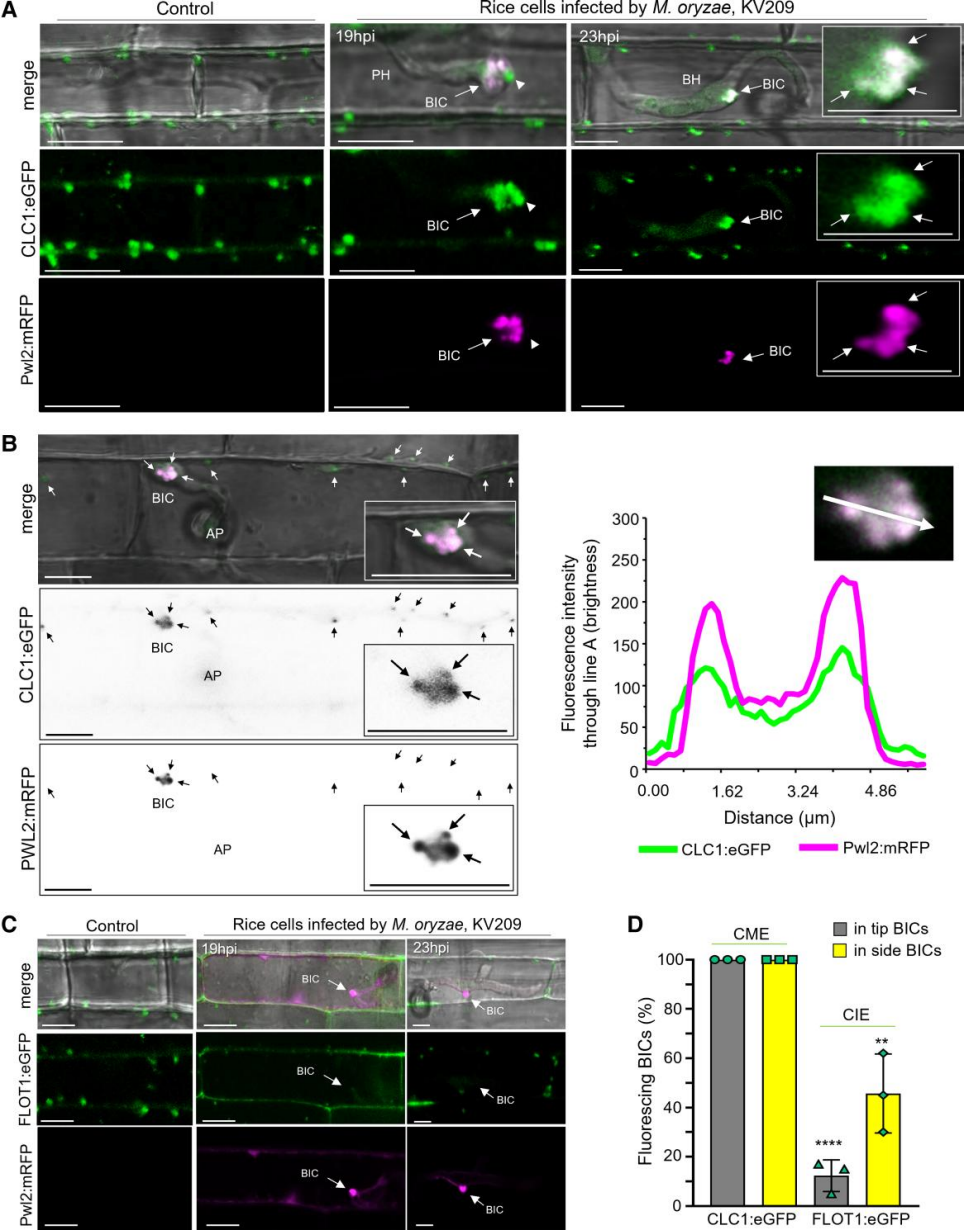
MECs colocalize with fluorescent CLATHRIN LIGHT CHAIN 1. **A)** Rice CME component OsCLC1:eGFP (green) colocalizes with individual MECs labeled with Pwl2:mRFP (magenta) in transgenic leaf sheaths. Shown left to right: noninoculated control; a cell with a primary hypha (PH) and a tip-BIC; and a cell with a bulbous IH (BH) and side-BIC. Colocalization is indicated by arrows in the inset. A MEC visualized in the tip-BIC by OsCLC1:eGFP (arrowhead in the 19 hpi image) lacks Pwl2:mRFP, consistent with separated effector fluorescences in some MECs in tip-BICs. **B)** Correspondence between OsCLC1:eGFP (green) and Pwl2:mRFP (magenta) in MECs, indicated by a fluorescence intensity linescan along the path of the white arrow. Images (top to bottom) are merge (brightfield with green and red fluorescence), and individual black and white inverse images of OsCLC1:eGFP and Pwl2:mRFP fluorescence. This image has been extracted from Supplemental Movie 3. **C)** Rice CIE-associated OsFLOT1:eGFP generally fails to colocalize with MECs (strain KV209 expressing Pwl2:mRFP) in transgenic leaf sheaths. **D)** Bar charts quantifying OsCLC1:eGFP or OsFLOT1:eGFP fluorescence in BICs, based on means of combined data from 3 biological replicates (data points show the means of the individual reps); error bars indicate standard deviation; 3 times 97 BICs observed each treatment. ****P < 0.0001; **P = 0.002. Scale Bars = 10 µm.

CME is the major endocytosis mechanism in plants, with some contribution by clathrin-independent endocytosis (CIE) (Chen et al. 2011; Ewers and Helenius 2011; Fan et al. 2015; Narasimhan et al. 2020). To assess the colocalization of endocytosis components with BICs, we generated transgenic rice lines expressing fluorescent fusion proteins associated with CME or CIE. For CME, we generated a C-terminal translational fusion of *eGFP* with the rice gene for *CLATHRIN LIGHT CHAIN 1* (*OsCLC1*), which encodes the subunit that interacts with clathrin heavy chains to form the coats in CCVs. Rice lines expressing OsCLC1:eGFP showed typical clathrin foci (Konopka et al. 2008; Narasimhan et al. 2020) in uninfected rice cells (Fig. 6). We also generated a C-terminal translational fusion of *eGFP* to the rice gene for *FLOTILLIN 1* (Os*FLOT1*), a lipid raft component involved in 1 particular form of CIE in Arabidopsis (*Arabidopsis thaliana*) (Otto and Nichols 2011; Li et al. 2012; Cao et al. 2020). We used the transgenic rice lines for leaf sheath assays with an *M. oryzae* strain expressing Pwl2:mRFP to label BICs.

In addition to typical clathrin foci around the cell periphery, all BICs contained fluorescence from OsCLC1:eGFP that colocalized with Pwl2:mRFP (Fig. 6, A and B and D). Indeed, clathrin fluorescence colocalized with individual MECs containing Pwl2:mRFP, Bas1:mRFP, or Bas170:mRFP fluorescence in both tip- and side-BICs (Fig. 6, A and B; Supplemental Fig. S6; Supplemental Movie 3). By contrast, very few (∼4%) hyphal tip-BICs colocalized with OsFLOT1:eGFP. Some colocalization of OsFLOT1:eGFP and Pwl2:mRFP occurred during later cell invasion stages in side-BICs beside mature bulbous hyphae (Fig. 6, C and D. The colocalization results from independent experiments are consistent with the notion that CME, but not OsFLOT1-associated CIE, plays a major role in the internalization of cytoplasmic effectors.

### Silencing of rice CME component genes blocks infection and MEC formation

**Figure 7.**
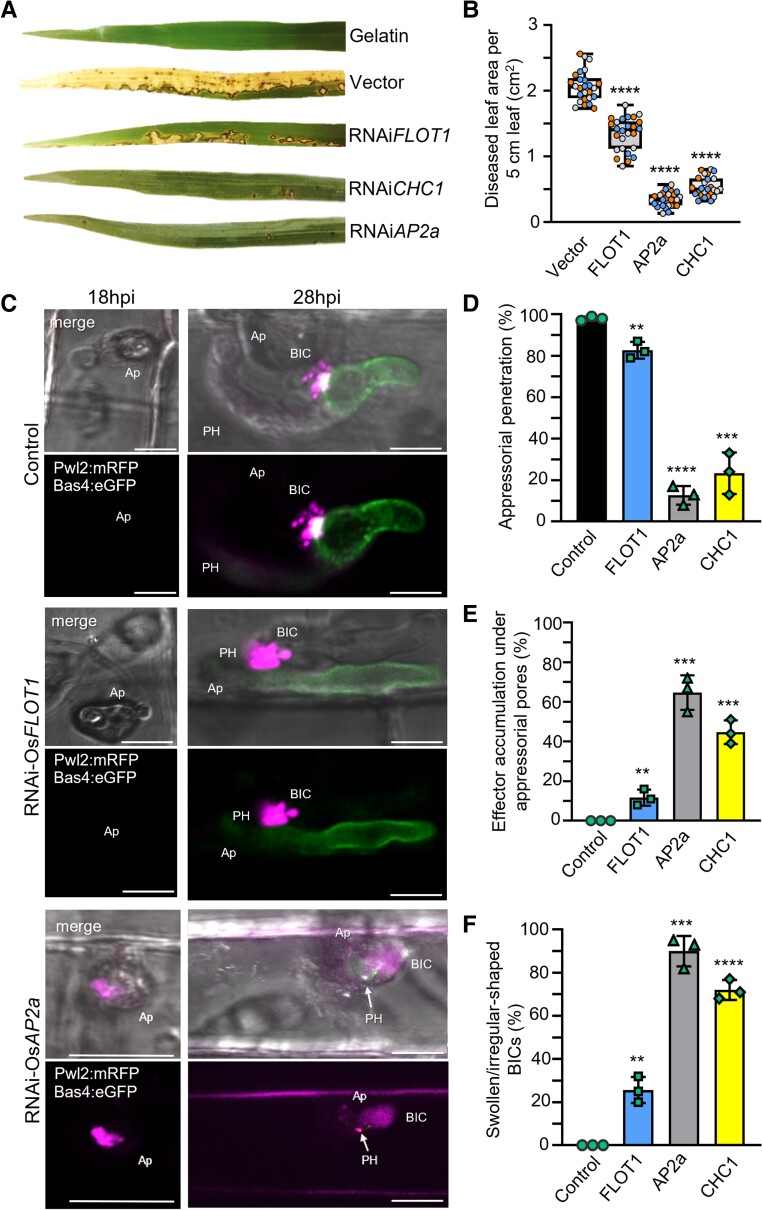
Silencing of CME genes suggests a role in effector translocation. **A)** Whole plant spray inoculation assays with *M. oryzae* strain Guy11 showed that VIGS of CME component genes *OsAP2*a and *OsCHC1* resulted in near total loss of pathogenicity compared to empty vector control plants. Silencing of *OsFLOT1* resulted in fewer, smaller lesions compared to the controls. **B)** Quantification of diseased area caused by strain Guy11 in 5 cm leaf segments of rice expressing RNAi*AP2a,* RNAi*CHC1*, RNAi*FLOT1* or empty vector. Box and whisker plots with individual data points are shown; data points in different colors represent different biological replicates. *P* < 0.0001 for all treatments; *n* = 9 rice plants per replication. **C)** Rice leaf sheath assays with strain KV217 (expressing Pwl2:mRFP and Bas4:eGFP) showed 3 phenotypes in the RNAi*AP2*a silenced rice compared to control and RNAi*FLOT1* rice. These included reduction in appressorial (Ap) penetration; accumulation of Pwl2:mRFP fluorescence under Ap at 18 hpi; and formation of short primary hyphae (PH), if any, with swollen BICs lacking punctate MECs at 28 hpi. Images (top: merged bright field, eGFP and mRFP fluorescence; bottom: eGFP and mRFP fluorescence) shown as projections of confocal optical sections. Scale bars = 10 µm. **D** to **F**) Quantification of appressorial penetration (**D**), effector accumulation under Ap (**E**), and swollen irregular BICs (**F**) in rice expressing RNAi*FLOT1*, RNAi*AP2*a, RNAi*CHC1* or the empty vector during infection by strain KV209 expressing Pwl2:mRFP (∼28 hpi). Bar charts are based on means of all data from 3 biological replicates (data points show means of individual reps); error bars indicate standard deviation. (**D**: **P = 0.0031, ***P = 0.0002, ****P < 0.0001; **E**: **P = 0.0075, ***P = 0.0002; **F**: **P = 0.0018, **** < 0.0001; 3 times 100 infection sites counted per treatment).

We assessed the impact of inhibiting CME and OsFLOT1-associated CIE on BICs and MECs using the brome mosaic virus system (Sun et al. 2013) to silence rice genes for endocytosis components that were previously characterized in Arabidopsis. Specifically, we silenced 2 rice CME genes, the *ADAPTOR PROTEIN COMPLEX 2 subunit 2α* (*OsAP2*α) and *CLATHRIN HEAVY CHAIN 1* (*OsCHC1*) genes (Di Rubbo et al. 2013; Larson et al. 2017; Aniento et al. 2021) as well as the *FLOTILLIN 1* (*OsFLOT1*) gene, which has been implicated in CIE in Arabidopsis (Li et al. 2012). Silencing of both *OsAP2α* and *OsCHC1* resulted in a severe reduction in pathogenicity in whole plant spray inoculation assays (Fig. 7, A and B). However, as expected, silencing of these genes caused stunting and decreased plant health (Supplemental Fig. S7), and the levels of pathogenicity in blast disease are known to be reduced in unhealthy rice plants. More significant is the phenotype of pathogen blockage at individual infection sites. Silencing of either CME component (RNAi-*AP2*α or RNAi-*CHC1*) led to a distinctive swollen BIC phenotype, with enlarged, abnormally shaped BICs that lacked punctate MECs (Fig. 7, C to F). The swollen BIC phenotype is consistent with the notion that effectors were still being secreted by the fungus but remained trapped within the EIHM due to the blockage of CME. By contrast, silencing of the *OsFLOT1* gene led to less severe stunting (Supplemental Fig. S7), had less of an impact on pathogenicity (Fig. 7, A and B), and had a minor impact (if any) on MEC formation (Fig. 7, C to F). For example, compare the images of BICs in control rice plants vs. RNAi-*FLOT1-*silenced and RNAi-*AP2*α*-*silenced plants in Fig. 7C at 28 h post inoculation (hpi), and the quantification of swollen BIC events in Fig. 7F.

In addition to swollen BICs at sites with primary hyphae, silencing of both CME genes decreased appressorial penetration and induced the accumulation of the cytoplasmic effector Pwl2:mRFP under failed penetration sites, where it is not normally observed (Fig. 7C-18hpi, D, E). By contrast, silencing of *OsFLOT1* had only minor effects on appressorium penetration and the accumulation of Pwl2:mRFP under failed appressoria. Similar to our finding of the early accumulation of Bas170 under appressoria before penetration, these results again suggest that effector uptake begins before host penetration. These results also suggest that CME plays a role in effector translocation at the appressorial penetration stage as well as from BICs.

### Chemical inhibition of the rice endocytosis machinery blocks *M. oryzae* infection

**Figure 8.**
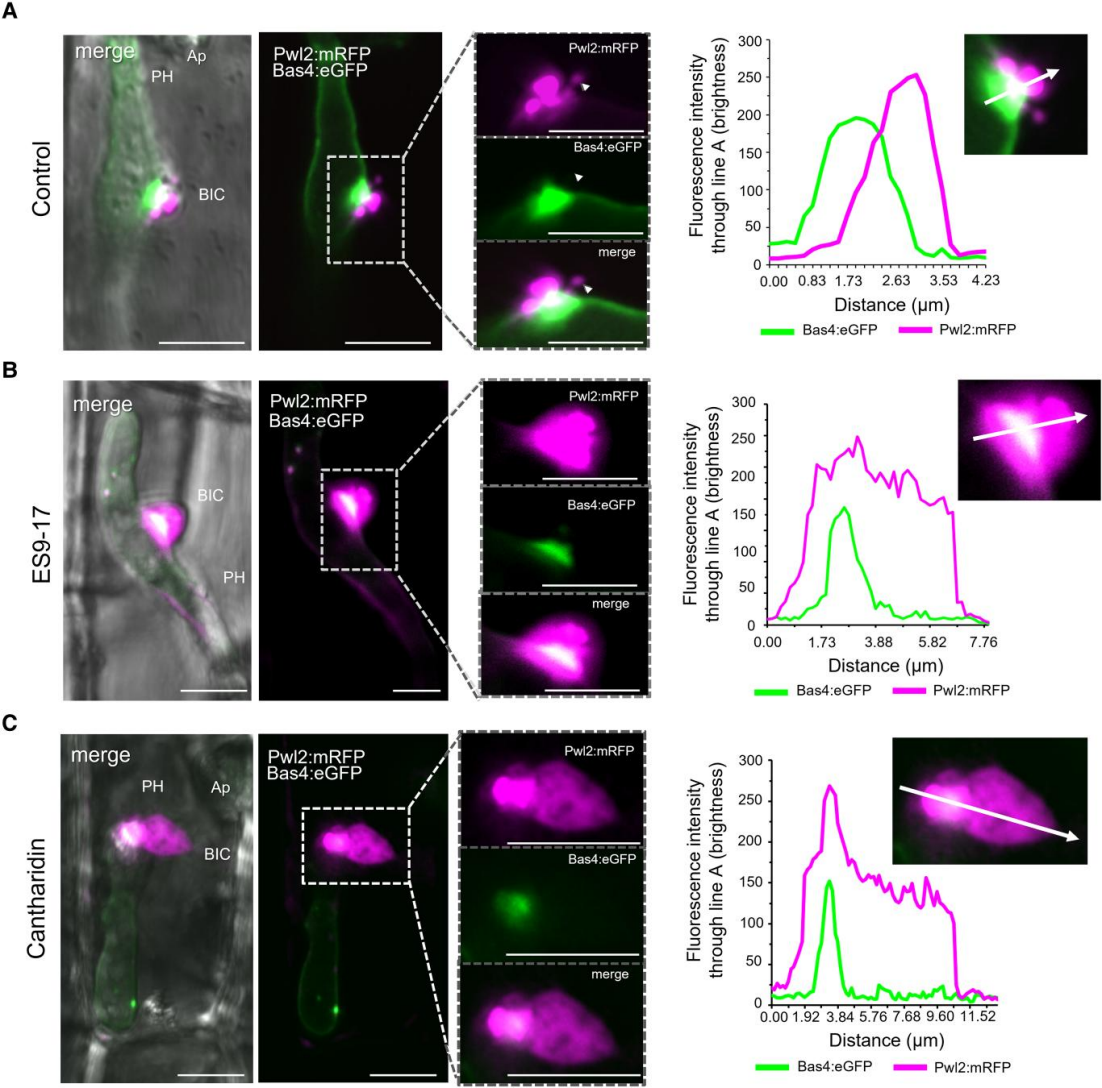
Treatment with CME inhibitors impacts localization of the cytoplasmic effector, but not the apoplastic effector. All panels show side-BICs formed during rice leaf sheath cell invasion by strain KV217 expressing Pwl2:mRFP and Bas4:eGFP. Relative localizations are shown by fluorescence intensity linescans for GFP (green) and mRFP (magenta) along the path of the white arrow. Inhibitor treatments were initiated at 26 hpi. **A)** In the untreated control, apoplastic effector Bas4:eGFP identifies an inner base layer in the BIC relative to the outer MEC structure for cytoplasmic effector Pwl2:mRFP. One MEC appears separated from the BIC (arrowhead in insets). **B** and **C**) Treatment with CLATHRIN HEAVY CHAIN inhibitor ES9-17 (**B**) or with CME signaling inhibitor cantharidin (**C**) results in swollen, irregular localization of Pwl2:mRFP in BICs lacking obvious MEC puncta. Bas4:eGFP localization appears unaffected. From left to right, images show merged bright field, eGFP and mRFP fluorescence; merged eGFP and mRFP fluorescence; insets with enlarged mRFP, eGFP, and merge views; and intensity line scans along the paths of the white arrows in insets. Images are projections of confocal optical sections. Ap, appressorium; PH, primary hypha. Scale bars = 10 µm.

Pharmacological approaches have enhanced studies in diverse biological systems (Robinson et al. 2008; Hicks and Raikhel 2010; Grassart et al. 2014; Fan et al. 2015). To further assess the potential role of the plant endocytic machinery in cytoplasmic effector translocation, we tested a series of chemicals that are reported to inhibit plant CME or CIE (Table 1). Compared with the nontreated control (Fig. 8A), treatment with the CME inhibitor Endosidin9-17 (ES9-17), which directly inhibits CLATHRIN HEAVY CHAIN function in both Arabidopsis and human cells (Dejonghe et al. 2019), led to the formation of abnormally shaped swollen BICs that lacked punctate MECs (Fig. 8B). This phenotype resembles the phenotype seen with the silencing of CME genes *OsCHC1* and *OsAP2α* (Fig. 7, C and F). Treatment with cantharidin, which inhibits signaling associated with CME, also produced swollen BICs lacking MECs (Fig. 8C). Fluorescence intensity line scans show distances that are 2.5 to 3-fold greater for Pwl2:mRFP after ES9-17 and cantharidin treatments with little (if any) impact on the localization of Bas4:eGFP in the base of the BIC (Fig. 8).

**Table 1.** Chemical inhibitors of endocytosis

Chemical	Target	Mode of action	Comments	References
Filipin	CIE	Binds sterols in membranes.	Toxic at higher concentrations; some impact on CME.	Rodal et al. 1999; Dutta and Donaldson 2012
Methyl-β-cyclodextrin	CIE	Depletes cellular membranes of sterols by increasing the water solubility of the sterol.	Caveolae-dependent endocytosis (animals); FLOT1-associated endocytosis (plants); some impact on CME.	Rodal et al. 1999; Dutta and Donaldson 2012; Li et al. 2012
Chlorpromazine	CME	Translocates clathrin and AP2 from the cell surface to intracellular endosomes.	Also inhibits CIE in some cells.	Wang et al. 1993; Dutta and Donaldson 2012
Cantharidin	CME	Inhibits PP2A (protein phosphatase 2A)	Affects flg22-mediated FLS2 endocytosis by targeting signaling.	Serrano et al. 2007
Triclosan	CME	Inhibits PP2A (protein phosphatase 2A)	Affects flg22-mediated FLS2 endocytosis by targeting signaling.	Serrano et al. 2007
Wortmannin	nd^a^	Inhibits PI3Ks, blocks late trafficking to the vacuole, multivesicular bodies and endocytosis.	Late endosomes lose clathrin; Potential CME association.	Bright et al. 2001; Sachse et al. 2002; Robinson et al. 2008
Endosidin9	CME	Inhibits the function of Clathrin Heavy Chain; has proton translocator activity	CHC inhibition in Arabidopsis and Drosophila; causes cytoplasmic acidification	Dejonghe et al. 2019; Dejonghe et al. 2016
Endosidin9-17	CME	Inhibits the function of clathrin heavy chain (CHC)	Inhibitor of CHC function in Arabidopsis and human cells. More specific version of ES9 lacking cytoplasmic acidification activity.	Dejonghe et al. 2019
Concanamycin A	nd	Targets V-type ATPase and blocks trafficking at the trans-Golgi network, endosome acidification.	Induces acidification crucial for endocytic pathways.	Maranda et al. 2001; Robinson et al. 2008

nd, not determined.

**Figure 9.**
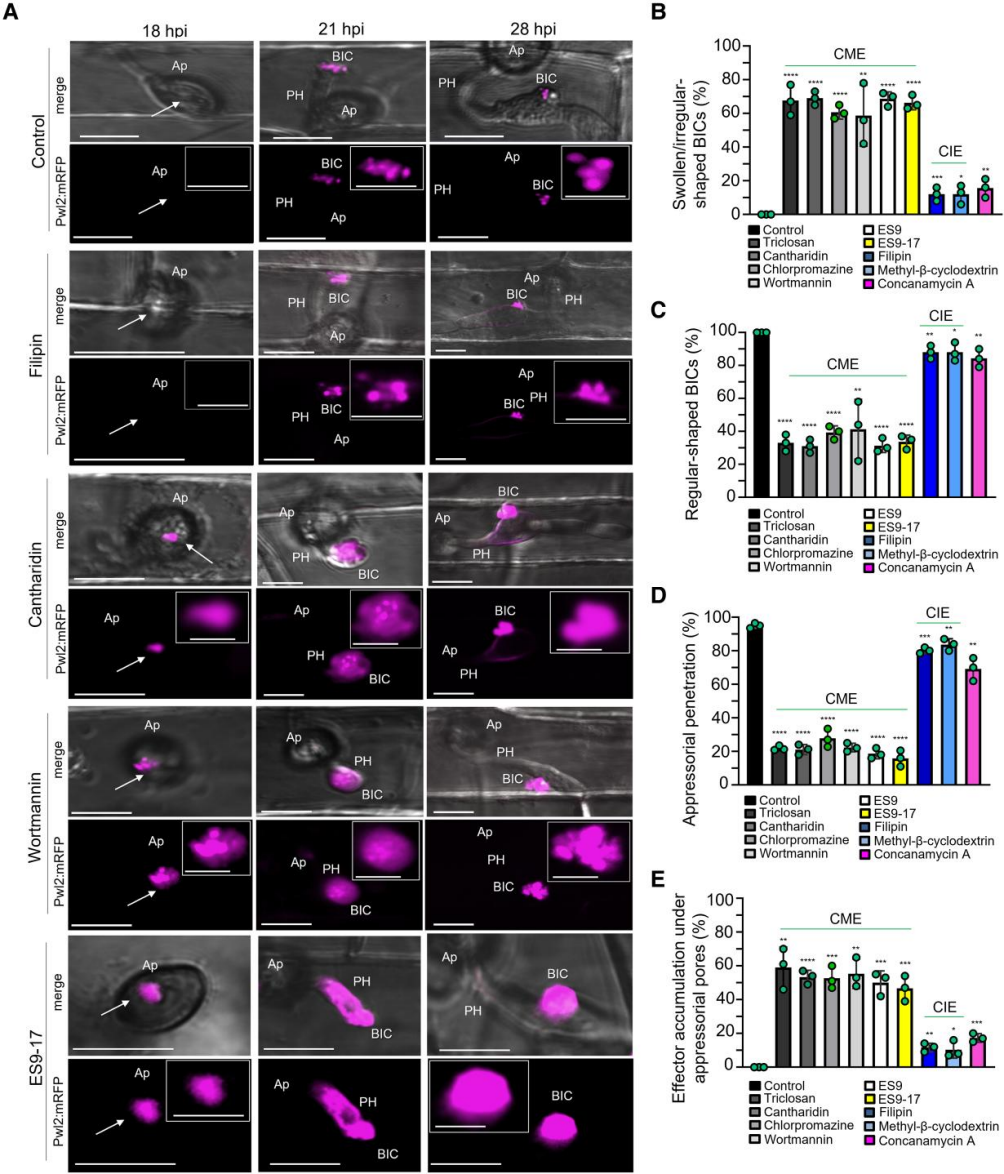
Chemical inhibition of rice CME impacts cytoplasmic effector localization in BICs and under appressoria. Effects of chemical inhibitors (Table 1) on localization of Pwl2:mRFP secreted by strain KV209 during invasion of rice sheath cells. Inhibitor treatments were initiated at 3 indicated time points. **A)** Images reflecting representative outcomes of inhibitor treatments. Compared to untreated controls and CIE-associated inhibitor filipin, the CME-associated inhibitors cantharidin, wortmannin, and endosidin9-17 (ES9-17) resulted in accumulation of effector fluorescence under appressoria (Ap, white arrows) at 18 hpi; in swollen tip-BICs on primary hyphae (PH) at 21 hpi; and in swollen side-BICs on bulbous IH at 28hpi. Images (bright field merged with mRFP above and mRFP alone below) are projections of confocal optical sections. Scale bars = 10 µm. Insets show enlarged views of the effector fluorescence with Scale bars = 5 µm. **B** to **E**) Quantification of swollen, irregular-shaped BICs (**B**), regular-shaped BICs (**C**), Ap penetration (**D**), and effector accumulation under Ap (**E**), in rice after treatment with 9 different inhibitors at 28 hpi. Bar charts are based on means of all data from 3 biological replicates (data points show the means of the individual reps); error bars indicate standard deviation. Three times 100 infection sites observed per treatment. In **B**: *P = 0.0202(MβC), **P = 0.0065(Filipin), **P = 0.0079(Con.A), **P = 0.005(Wort.), ****P < 0.0001; in **C**: *P = 0.02(MβC), **P = 0.0065(Filipin), **P = 0.0075(Con.A), **P = 0.005(Wort.), ****P < 0.0001; in **D**: *P* = 0.0068(MβC), **P = 0.0004(Filipin), **P = 0.0032 (Con.A), ****P < 0.0001; in **E**: *P = 0.0222(MβC), **P = 0.0013(Filipin), ***P = 0.0003(Con.A), ***P = 0.0002(ES9), ***P = 0.0003(ES9-17), **P = 0.0004(Wort.), **P = 0.0011(Tricl.), ***P = 0.0002(Chlorpr.), ****P < 0.0001(Canth.).

We quantified Pwl2:mRFP localization patterns during the infection of rice treated with 5 chemicals reported to affect CME, 2 chemicals reported to affect CIE, and 2 chemicals with less defined roles in inhibiting endocytosis (Fig. 9). The 5 CME inhibitors included ES9-17 and cantharidin (Fig. 8) along with Endosidin9 (ES9), chlorpromazine, and triclosan (Table 1). Compared to the untreated controls, treatment with each of these 5 CME-associated inhibitors generated a high proportion of swollen, irregularly shaped BICs lacking punctate MECs (Fig. 9, A to C). By contrast, treatment with the CIE inhibitors filipin and methyl-β-cyclodextrin had only minor to no effects on BIC effector fluorescence patterns (Fig. 9, A to C). Two chemical inhibitors potentially associated with CME (Table 1) had different impacts on BIC effector fluorescence patterns. Wortmannin inhibits phosphatidylinositol 3-kinase (PI 3-kinase) activity and is implicated in late endosomal trafficking (Robinson et al. 2008). Wortmannin treatment produced the swollen BIC phenotype. By contrast, concanamycin A, which inhibits vacuolar-type ATPase activity and induces vacuolar acidification (Robinson et al. 2008), had little impact on BIC structure (Fig. 9, A to C).

Moreover, treatment with the CME inhibitors triclosan, cantharidin, chlorpromazine, ES9, and ES9-17, but not with the CIE inhibitors filipin and methyl-β-cyclodextrin, resulted in reduced appressorial penetration and the abnormal accumulation of the cytoplasmic effector Pwl2:mRFP under appressorial penetration sites, where it is not normally observed (Fig. 9A—compare 18 hpi samples treated with CIE and CME inhibitors; Fig. 9, D and E). Wortmannin, which produces swollen BICs, also affected appressorial function and resulted in abnormal Pwl2:mRFP accumulation under appressoria (Fig. 9, A and D and E). Concanamycin A produced none of the CME-associated phenotypes (Fig. 9). Therefore, the chemical inhibition of endocytosis in rice cells undergoing *M. oryzae* infection mimicked the results obtained by VIGS, further supporting a role for CME in effector uptake into rice cells. These results using CME inhibitors, together with the results using Bas170:mRFP (Fig. 1, D to F), support the notion that effector uptake begins before or at the point of host penetration.

## Discussion

**Figure 10.**
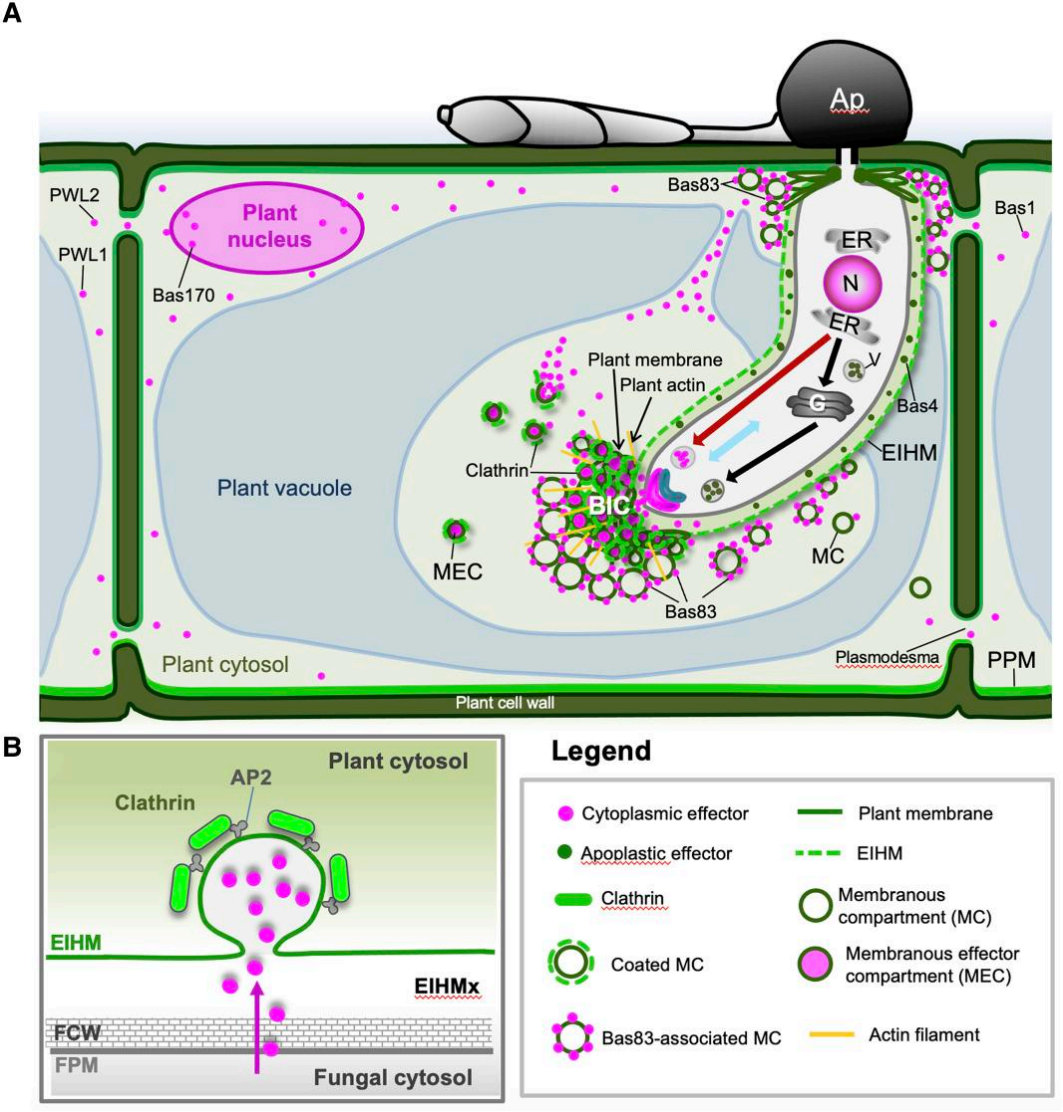
Model for the translocation of *M. oryzae* cytoplasmic effectors into rice cells. **A)** A tubular primary hypha (PH) is depicted growing immediately after appressorial penetration but before differentiation to the first bulbous IH cell. The blast fungus uses the conventional BFA-sensitive pathway to secrete apoplastic effectors such as Bas4 (green dots), which remain trapped inside the EIHM (green dashed line) enclosing the PH. Cytoplasmic effectors are secreted via a nonconventional BFA-insensitive pathway and accumulate in the BIC. Our current study shows that secreted fluorescently-labeled cytoplasmic effectors (magenta dots) are packaged in dynamic vesicle-like MECs that colocalize at the BIC interface with fluorescent rice plasma membrane (LTi6B:GFP) and with fluorescent rice CLATHRIN LIGHT CHAIN (OsCLC1:eGFP). Two newly characterized cytoplasmic effectors have distinct localization patterns. Bas170 localizes to MECs beneath appressoria and in host nuclei before penetration. Bas83 localizes to distinct membranous compartments (MCs) lacking effector fluorescence, possibly recruiting host membrane fragments to a region of active endocytosis. Cytoplasmic connections between BICs and the host cell periphery facilitate rapid spread of translocated effectors. Key: In the fungus: N, nucleus; ER, endoplasmic reticulum; G, Golgi apparatus; V, transport vesicle; black arrow, golgi-dependent secretion of apoplastic effectors; red arrow, golgi-independent secretion of cytoplasmic effectors; blue curved structure, exocyst complex components Exo70 and Sec5; magenta curved structure, SNARE complex component Sso1. In the plant: PPM, plant plasma membrane. **B)** Working model for MEC formation via host CME based on blocking of MEC formation via VIGS targeting of CME components OsCHC1 and OsAP2α, and on studies with multiple chemical inhibitors associated with endocytosis. AP2, adaptor protein-2 complex; EIHMx, extrainvasive hyphal matrix; FPM, fungal plasma membrane; FCW, fungal cell wall.

The rice blast fungus colonizes living rice cells in the form of specialized intracellular IH that flood invaded host cells with translocated cytoplasmic effectors, including effectors that move into the surrounding cells before pathogen invasion (Khang et al. 2010; Mochizuki et al. 2015; Sakulkoo et al. 2018; Jones et al. 2021). Our current results provide strong evidence that the blast fungus coopts CME in the host to translocate cytoplasmic effectors at the specialized interfacial region known as the BIC. We describe fungal-induced vesicle-like MECs as dynamic membrane-bound cytoplasmic effector-containing bodies in the BIC and occasionally in the host cytoplasm. Individual MECs colocalized with fluorescently labeled rice plasma membrane (Fig. 3C) and with fluorescently labeled rice CLATHRIN LIGHT CHAIN, (Fig. 6, A and B; Supplemental Fig. S6; Supplemental Movie 3). VIGS of genes encoding 2 rice CME components, CLATHRIN HEAVY CHAIN 1 (OsCHC1) and ADAPTOR PROTEIN COMPLEX subunit 2α (OsAP2α), decreased pathogenicity and resulted in a swollen BIC phenotype in which cytoplasmic effectors appeared to be retained inside an enlarged BIC compartment lacking punctate MECs (Fig. 7). Additionally, treatment with 6 different CME-associated pharmacological agents, including CLATHRIN HEAVY CHAIN inhibitor Endosidin9-17 (Dejonghe et al. 2019), resulted in the swollen BIC phenotype. Various experiments included the retention of the fluorescent apoplastic effector Bas4:eGFP (Figs. 1, 7, and 8) inside the EIHM matrix and the exclusion of the endocytosis tracer dyes FM4-64 or FM1-43 from IH membranes (Fig. 3, D and E; Supplemental Movie S2) as controls for the integrity of the EIHM, across which translocation occurs. On another front, the use of the fluorescently labeled effector Bas83:mRFP identified a second type of fungal-induced plant membranous compartment surrounding BICs that could play a role in replenishing host membranes needed for extensive endocytic activity (Fig. 4; Supplemental Fig. S4). Taken together, our results provide evidence that BICs include a focused region of hijacked plant CME for internalizing cytoplasmic effectors inside living plant cells (Fig. 10).

Our results provide details on cytoplasmic effector dynamics during the early biotrophic stage of blast disease, as defined by Jones et al. (2021). For initially invaded cells, early biotrophy includes appressorial penetration of the plant surface and differentiation into intracellular filamentous primary hyphae (Fig. 10). We report that the effector Bas170:mRFP was present in punctate MECs beneath appressoria and in host nuclei even before primary hyphal growth (Fig. 1D). Fluorescent cytoplasmic effectors were packaged into dynamic MECs in tip-BICs at primary hyphal tips (Fig. 2A) at the same time as fluorescently labeled cytoplasmic effectors were observed inside the host cell (Supplemental Fig. S2; Supplemental Movie 1). Early biotrophy is characterized by the growth of the fungus inside a sealed EIHM compartment that retains secreted apoplastic effectors and by open plasmodesmata, enabling cell-to-cell movement of translocated effectors (Khang et al. 2010; Sakulkoo et al. 2018; Jones et al. 2021).

After the BICs moved beside the first differentiated bulbous IH cells, cytoplasmic effectors appeared in fewer, larger MECs, and a few MECs were observed in the cytoplasm near side-BICs. This potentially indicates a slow-down in MEC formation, and perhaps a slow-down in the mechanism for effector release from MECs, which is consistent with the previous finding that cytoplasmic effector secretion slows and stops after the further growth of bulbous IH cells. Indeed, very large effector-labeled puncta were rarely observed in some host cells at later stages of invasion (Fig. 2C). Active BICs are surrounded by host cytoplasm with dynamic connections to the cell periphery (Khang et al. 2010; Supplemental Movie 1), where cytoplasmic streaming would rapidly disperses effectors to plasmodesmata. The late stage of biotrophy (occurring by the 21-hyphal cell stage) involves continued IH growth after apoplastic effectors are released and retained in the invaded cell via the disruption of the EIHM and closure of plasmodesmata (Khang et al. 2010; Jones et al. 2021). The host vacuole remains intact but shrinks as IH continue growing. Finally, at around the 28-hyphal cell stage, both the host plasma membrane and vacuolar membrane become disrupted, indicating death of the invaded cell (Mochizuki et al. 2015; Jones et al. 2021). At that time, multiple IH move into neighboring cells, with each hypha reestablishing early biotrophy and the secretion of cytoplasmic effectors into BICs.

The size differences between MECs and plant CCVs suggests that plant CME is modified by the fungus (Fig. 2). As measured by fluorescence microscopy, MECs have a median diameter of 249 nm in tip-BICs, and they increase in size to a median diameter of 647 nm in side-BICs (Fig. 2D). These results confirm and expand on the previous finding that cytoplasmic effectors occur in ∼500 nm puncta in side-BICs (Nishizawa et al. 2016). By contrast, plant CCVs have mean diameters of 59.1 nm for the pentagonal basket population, 66.1 nm for the hexagonal basket population, and 85 nm for the irregularly ordered basket population (Narasimhan et al. 2020). The clear increase in MEC sizes through time could be due to the aggregation and/or fusion of MECs. Narasimhan et al. (2020) reported that Arabidopsis CCVs show delayed and sequential uncoating, and they presented evidence for aggregated, partially fused CCVs associated with internal membranes. The next steps in this analysis should include electron microscopy for detailed structural analysis of MECs.

CIE, involving sterol-rich membrane microdomains or lipid rafts, is less understood in plants than CME (Aniento et al. 2021). The pharmacological inhibitors filipin and methyl-ß-cyclodextrin, which bind sterols or deplete membranes of sterols, respectively, are mainly associated with CIE. In contrast to treatments with known CME inhibitors, filipin and methyl-ß-cyclodextrin had little or no impact on MEC structure and pathogenicity (Fig. 9). The Arabidopsis membrane microdomain-associated protein FLOTILLIN 1 was shown to be internalized in a type of CIE and to be required for seedling development (Li et al. 2012). Fluorescently labeled OsFLOT1:eGFP rarely localized to BICs (Fig. 6C). Silencing of the rice *OsFLOT1* gene resulted in stunted seedling growth (Supplemental Fig. S7) but had minimal effects on MEC formation (Fig. 7). However, our results with CIE-associated inhibitors do not rule out a minor role for CIE in effector translocation because inhibiting CIE did have some effect. It remains to be determined if this effect is due to a minor role for CIE or to the reported minor impact of the CIE inhibitors filipin and methyl-ß-cyclodextrin on CME (Table 1; Rodale et al. 1999; Dutta and Donaldson 2012).

Data from both VIGS and pharmacological inhibitor studies could potentially be misinterpreted. Rice plants that received the *OsCHC1* and *OsAP2α* silencing vectors showed significantly reduced rice blast symptoms compared to the empty vector control and to *OsFLOT1*-silenced lines (Fig. 7; Supplemental Fig. S7). However, this could be due (at least in part) to inevitable health costs to the plant through silencing of genes involved in essential cellular processes. Unhealthy rice plants are generally less susceptible to blast disease than healthy plants (Kato 1974). Additionally, care must be taken in the interpretation of results from pharmacological studies based on evidence that endocytosis inhibitors characterized in mammalian systems may not have the same targets in plants (Dejonghe et al. 2016; Dejonghe et al. 2019) and that pharmacological agents also have off-target effects (Hicks and Raikhel 2010; Dutta and Donaldson 2012; Dejonghe and Russinova 2017). However, the consistent swollen BIC phenotype observed only in plants under treatments that inhibit CME, which are consistent with the inhibition of MEC formation (Figs. 7 to 9), lends credibility to our interpretation of these results.

Effector localization dynamics point to the fine-tuned staging of expression and secretion of *M. oryzae* effectors, including before appressorial penetration. This is similar to the reported waves of effector expression in the crucifer pathogen *Colletotrichum higginsianum*, including the secretion of ChEC effectors under the appressorial pores before the penetration of Arabidopsis cells (Kleemann et al. 2012). These observations are consistent with the finding that rice cells recognize and respond to *M. oryzae* before and independently of appressorial penetration (Xu et al. 1998), as well as the hypothesis that the *M. oryzae* appressorial pore, a transient cell wall-less region of the appressorium adjacent to the plant cuticle, might be involved in molecular communication between the pathogen and host before penetration (Howard and Valent 1996). The cytoplasmic effector Bas170, which naturally accumulates in host nuclei before the visible growth of primary hyphae (Fig. 1D), joins the presumed secondary metabolite produced by the *AVR* gene *ACE1* (encoding a hybrid between a polyketide synthase and a nonribosomal peptide synthetase) (Fudal et al. 2007; Collemare et al. 2008) as a candidate for secretion through the appressorial pore. The effector Pwl2:mRFP, which is not normally observed before the tip-BIC stage, accumulated under appressoria that failed to penetrate after both VIGS silencing and chemical inhibition of rice CME, again highlighting a potential role for fungal appressoria in effector delivery. Recently, Yan et al. (2023) classified 10 modules of temporally coexpressed genes ranging from 0 hpi to 144 hpi, including some localized to the appressorial pore, among 546 predicted *MEP* (*Magnaporthe* effector protein) genes.

There is evidence supporting occurrence of CME at the EHM in a rust fungal pathosystem. That is, transmission electron micrography of the interfacial matrix between the intracellular hyphae [called monokaryotic (M)-haustoria based on their functional analogy to dikaryotic haustoria] of the monokaryotic rust fungus *Uromyces vignae* and its host cowpea (*Vigna unguiculata*) revealed tubular-coated pits on the EHM at M-haustoria hyphal tips (Stark-Urnau and Mendgen 1995). Long tubules extending from the EHM into the host cytoplasm, as well as coated vesicles in the surrounding host cytoplasm, were labeled with an antibody recognizing the clathrin heavy chain subunit, indicating that CME occurs at the EHM (Stark-Urnau and Mendgen 1995). It was suggested that these endocytic vesicles might be involved in effector uptake into host cells in addition to membrane recycling from host exocytosis (Stark-Urnau and Mendgen 1995; O'Connell and Panstruga 2006). Unfortunately, the lack of transformation capability and the lack of identified effectors make it difficult to follow up on these intriguing findings with *U. vignae*. Additional studies supported our finding that inhibiting lipid raft-mediated endocytosis by filipin and methyl-β-cyclodextrin treatments had little effect on BIC structure and MEC formation. Freeze-fracture transmission electron microscopy following filipin treatment revealed an absence of granular filipin-sterol complexes on the EHM of 2 rust fungi, *Puccinia coronata* and *U. appendiculatus* (Harder and Mendgen 1982), suggesting that the EHM contains less sterol than normal plasma membranes and that the EHM appears to be depleted in sterol-rich lipid rafts.

The notion that different infection strategies are employed among various filamentous biotrophic or hemibiotrophic pathogens is consistent with the use of different strategies for effector translocation in different pathosystems. A recent report indicated that a complex of 7 proteins from the smut pathogen *U. maydis* is critical for infection and is implicated in the translocation of cytoplasmic effectors into maize cells (Ludwig et al. 2021). This basidiomycete fungus colonizes host tissue as hyphae that pass through and between host cells without obviously specialized interfacial zones. Fluorescent fusion proteins of cytoplasmic effectors have not been visualized in the host cell cytoplasm in this pathosystem (Lo Presti et al. 2017). *U. maydis* produces galls on maize seedlings and grows systemically as the plant grows to finally invade ovaries, which swell into dramatic sporulating galls. By contrast, *M. oryzae* densely colonizes localized tissue areas that will become visible eyespot lesions, releasing spores to reinitiate the 7-day infection cycle. Different pathogenic lifestyles could present different demands for cytoplasmic effector translocation.

Taken together, our data strongly suggest that the blast fungus co-opts CME for the internalization of *M. oryzae* effectors inside living rice cells, beginning even before appressorium penetration of the host surface. Further confirmation will come through continuing research to determine how the endocytic machinery is recruited to BICs and how it interacts with effector cargos (Qi et al. 2018). It is important to understand if (and if so, how) Bas83 contributes to recruitment of BICs to the membrane and EIHM surrounding BIC-associated cells. It is of interest to understand how the supposedly coordinately expressed effectors Bas1:eYFP and Pwl2:mRFP can be sorted into different MECs, as seen in Fig. 2A. It is important to understand the structure and dynamics of MECs, including why MECs become larger and more persistent as BICs mature (Fig. 2). Identifying effectors with critical roles in the translocation process is a high priority, including effectors involved in disrupting MECs and releasing effectors into the rice cytoplasm. It is important to understand the role of appressoria in effector secretion before penetration and how these early effectors, such as the host-nucleus-localized effector Bas170, promote infection. Finally, it is critical to translate the molecular mechanisms of the biotrophic invasion of blast fungi into strategies for controlling devastating diseases in rice, wheat and other food crops worldwide.

## Materials and methods

### Live cell imaging of *M. oryzae* effectors *in planta*

Fungal strains (*M. oryzae*) were stored on dried filter papers at −20˚C and cultured on rice polish or oatmeal agar agar plates (Valent et al. 1991; Oliveira-Garcia and Valent 2021) at 25°C for up to 2 wk under continuous light in Percival Scientific (Model CU-36L4) tissue culture incubators equipped with one half fluorescent lights (FT20T12/cw, 20W) and one half black lights (FT20T12/BL, 20W). Rice (*O. sativa*) plants for both whole plant spray inoculation and detached leaf sheath assays were grown in Baccto Top Soil (Michigan Peat Co., Houston, Texas) in a Conviron BDW120 growth room with equal numbers of halogen lamps (Philips 409821, 72 watt replacement for 100 Watt incandescent bulbs) and metal halide lamps (Philips ED37, 400 W). At rice seedling height, ∼1 m from the bulbs, light ranged in intensity from 600 to 700 µmol m^−2^ s^−1^. Plants were grown at 80% relative humidity under a daily cycle of 12 h of light at 28°C and 12 h of darkness at 24°C. At 2 wk, plants are fertilized with Jack's Professional Peat Lite 20-10-20 Fertilizer (#77860; JR Peters, Inc.; Allentown, PA). Whole plant infection assays were performed by spray inoculation of 2 to 3-week-old rice plants as described (Valent et al. 1991).

Rice leaf sheath inoculations were performed as described (Kankanala et al. 2007) with the following modification. We used sheath pieces that were thickly trimmed (∼7 rice cell layers thick) compared to thinner trimmed sheaths (∼3 cell layers thick) in previous publications. Brightfield images were less detailed, but the endocytic machinery appeared to be more active, providing enhanced microscopic views of fluorescent marker dynamics. Susceptible rice variety YT-16 was used unless otherwise mentioned. Briefly, 7-cm long leaf sheath pieces from 3-week-old plants were placed in a sealable Pyrex glass moist chamber. Leaf sheath sections were placed on inverted 8-well PCR tube strips to avoid contact with wet paper and to hold the epidermal cells directly above the mid-vein horizontally flat for the uniform distribution of inoculum in trimmed leaf sheath pieces (Oliveira-Garcia and Valent 2021). A spore suspension (10^4^ spores/ml in sterile 0.25% gelatin, Cat. #G-6650, Sigma-Aldrich) was prepared from 10-day-old cultures and injected into one end of the sheath using a 100-ml pipette. Each segment was trimmed at 18 to 30 h post inoculation (hpi), treated with specific dyes or inhibitors, or imaged immediately by laser confocal microscopy. Biological replicates were independent experiments performed with fungal cultures fresh out of frozen storage and with new rice plants. All conclusions are supported by at least 3 biological reps, with each replication including observation of ∼100 infection sites.

Confocal imaging was performed with a Zeiss LSM780 confocal microscope system using 2 water immersion objectives, C-Apochromat 40x/1.2 WCorr. and C-Apochromat 63x/1.2WCorr. Excitation/emission wavelengths were 488 nm/505 to 550 nm for eGFP and FM1-43 and 543 nm/560 to 615 nm for mRFP, mCherry, Phalloidin and FM4-64. Image acquisition and processing, some MEC quantification, and fluorescence intensity line scans were generated using Zeiss ZEN 2010 software. Images of MECs in Fig. 3C were obtained using a Leica SP8 confocal microscope system with a water immersion objective (HC PL APO 63x/1.20 WCorr). Excitation/emission wavelengths were 488 nm/505 to 550 nm for eGFP and 543 nm/560 to 615 nm for mRFP. Leica SP8 software at the University of Exeter and at the Louisiana State University Agricultural Center was used for some MEC quantification and for analyzing ES9 and ES9-17 CME inhibitor assays.

### Size measurements of fluorescent MECs

To measure MEC sizes, high-quality confocal microscopy images of BICs were generated from YT16 rice undergoing infection by *M. oryzae* strains KV170 or KV209 expressing Bas1:mRFP or Pwl2:mRFP, respectively. We began with maximum intensity projections of Z-stack image series of BICs obtained under Zeiss and Leica confocal microscopes. However, when MECs were overlapping in the collapsed images, we attempted to distinguish between 2 or more MECs using individual images in the Z-stack. For example, see Supplemental Movie 1. Scale bars were set to 1 µm. Images focused on regions containing MECs were opened in ImageJ (Schneider et al. 2012), and MEC diameters were measured using the length measurement tool. Specifically, distances in pixels were correlated to the number of pixels in 1 µm (1000 nm) scale bars and converted to nm. Data were exported in Excel and analyzed using Prism 9 software.

### Fungal strains, DNA manipulation, and fungal transformation

*M. oryzae* wild-type strain Guy11, a field isolate from rice in French Guiana, was obtained from J.L. Notteghem (Centre de Cooperation Internationale en Recherche Agronomique pour le Developpment, France). *M. oryzae* transformants are described in Supplemental Table S1. Effector:mRFP and Effector:eGFP expression plasmids were constructed by PCR amplifying different effector gene regions and cloning them for in frame expression of C-terminal mRFP or eGFP. Details of the construction of each plasmid are described in Supplemental Table S2. All primers used are listed in Supplemental Table S3. For all fusion constructs, transcriptional and translational fusions were verified by DNA sequencing. Plasmids were transformed into Guy11 using *Agrobacterium tumefaciens*–mediated transformation (Khang et al. 2006). In several cases, 2 fluorescently labeled effectors were introduced by cotransformation with separate plasmids (Sweigard et al. 1995). For each plasmid construct, 10 independent transformants were assayed for fluorescence intensity during host invasion and for consistent localization patterns. At least 2 independent transformants with high fluorescence intensities and identical sporulation, growth and infection phenotypes to the wild-type were stored and studied further (see Supplemental Table S1).

### Strategies for the targeted deletion of *BAS83*

#### Split marker recombination method for BAS83 gene knockout via protoplast transformation

Targeted gene replacement mutation of the *M. oryzae BAS83* gene was attempted using the split marker strategy as modified by Kershaw and Talbot (2009). Gene replacement was performed by replacing the 606-bp *BAS83* locus with a hygromycin resistance selectable marker encoding a 1.4-kb hygromycin phosphotransferase (*HPH*) resistance cassette (Carroll et al. 1994). The 2 overlapping segments of the *HPH* templates were PCR amplified using primers M13F with HY and M13R with YG (Catlett et al. 2003) (see Supplemental Table S3) as described (Kershaw and Talbot 2009). One kilobase pair DNA fragments upstream and downstream of the *BAS83* open reading frame were generated using the primers Bas83LF-F and Bas83LFHY-R and Bas83RFYG-F and Bas83RF-R amplified from genomic DNA of Guy11. A second-round PCR was performed to fuse the overlapping split *HPH* marker templates with the left and right *BAS83* flanking sequences. Guy11 was then transformed with the deletion cassettes (2 μg of each flank). Putative transformants were selected in the presence of hygromycin B (200 μg/ml) and analyzed by PCR using the primers Bas83:BASKOtest-F and Bas83:BASKOtest-R (Supplemental Table S3; Supplemental Fig. S5). This assay was repeated 3 times. Sequences on either side of *BAS83* were retrieved from the NCBI database (https://www.ncbi.nlm.nih.gov/).

#### Attempted BAS83 deletion via *Agrobacterium*-mediated transformation

For gene replacement transformation, cassettes were constructed by amplifying ∼1.0 kb of 5′- and 3′-flanking regions for the predicted coding sequence. The *HPH* gene (Carroll et al. 1994) was cloned between the flanking sequences using fusion PCR. The 3 pieces together were cloned first into the pJET1.2 vector (ThermoFisher Scientific) for sequence analysis and later into binary vector pGKO2 (Addgene: Plasmid #63617; Khang et al. 2005) using a restriction ligation strategy. Conidia from laboratory strains KU70 and KU80 (derivatives of Guy11 with deletions of MoKU70 and MoKU80 to enhance homologous recombination) were transformed using *A. tumefaciens* (Khang et al. 2006) strains AGL1 and EHA105 (Hellens et al. 2000). After 2 rounds of selection in TB3 medium containing 200 μg/ml of hygromycin, 400 independent fungal transformants were analyzed for gene replacement events by PCR amplification using the primers Bas83:BASKOtest-F and Bas83:BASKOtest-R (Supplemental Fig. S5). This assay was repeated 3 times.

### Staining with FM4-64, FM1-43, and rhodamine phalloidin and treatment with pharmacological inhibitors

To examine membrane dynamics at the BIC, rice sheaths (cv. YT16) were inoculated with *M. oryzae* transformants expressing fluorescently labeled effectors (2 × 10^4^ spores/ml in 0.25% gelatin solution) and incubated for 20 to 26 h at 25˚C for subsequent treatment with FM4-64 or FM1-43 (4 mg/ml in water). Aqueous 17 mM stock solutions of FM4-64 (Cat #13320, Invitrogen, Carlsbad, CA) and FM1-43 (Cat # T3163, Invitrogen, Carlsbad, CA) were prepared and stored at −20˚C as described (Kankanala et al. 2007). *In planta*, inoculated leaf sheaths at 24 h.p.i. were hand-trimmed as for microscopy and incubated in a 10-mM aqueous working solution for 3 to 5 h. Leaf sheaf sections were subsequently transferred to ultrapure water and incubated for 20 min (25°C) to remove excess dye. For *in planta* experiments with Rhodamine Phalloidin (Cat# R415, ThermoFisher Scientific), we prepared working solutions of 66 µM in 0.1% DMSO and treated the inoculated tissue as described for FM dyes.

To examine the effects of endocytosis inhibitors on *in planta* effector uptake, inoculated trimmed rice sheaths were incubated at 25°C with endosidin9 (ES9) (10 µM), endosidin9-17 (ES9-17) (30 µM), methyl-β-cyclodextrin (20 mM), chlorpromazine (10 µg/mL), cantharidin (25 mM), fluazinam (20 mM), triclosan (20 mM), wortmannin (20 nM), or concanamycin A (50 mM) (all from Sigma) solution for 1 to 5 h. Negative controls were performed with ultrapure water (methyl-β-cyclodextrin, chlorpromazine, and fluazinam) or 0.1% DMSO (ES9, ES9-17, cantharidin, filipin, and wortmannin) (see Table 1).

### Generation of transgenic rice plants

Production of transgenic plants expressing LTi6B:GFP (LOW-TEMPERATURE INDUCED PROTEIN 6B fused to GFP, a plasma membrane marker in rice) was previously described (Mentlak et al. 2012; Giraldo et al. 2013). Transformants of rice cultivar Sasanishiki expressing LifeAct:eGFP were generated in the laboratory of Nicholas J. Talbot. Plasmids for expressing fusions of the rice *OsCLC1* and *OsFLOT1* genes with *eGFP* using their native promoters were constructed by PCR amplifying the promoter and coding sequences and cloning them for in-frame expression with C-terminal eGFP. The *OsCLC1* gene was amplified from genomic DNA of YT16 rice using the primers KpnI_OsCLC1Prom1-F1 and XhoI_OsCLC1-R1 (see Supplemental Table S3). The PCR product was digested with *Kpn*I and *Xho*I and integrated into pSH1.6EGFP (pSH1.6EGFP was a gift from Holger Deising & Amir Sharon; Addgene plasmid # 42323; http://n2t.net/addgene:42323; RRID:Addgene_42323). The *OsFLOT1* promoter and coding sequences were amplified from genomic DNA from YT16 rice using the primers HindIII_OsFlot1Prom1-F1 and Eco47III_OsFlot-R1. The PCR product was digested with *Hind*III and *Eco*47III and integrated into pSH1.6EGFP. The *CLC1:EGFP* sequence was amplified from pSH1.6_CLC1:eGFP using the primers CACC_OsCLC1-F1 and eGFPT2-R2 and cloned into pENTR (pENTR™/D-TOPO™ Cloning Kit, ThermoFisher Scientific) following the manufacturer's protocol. *FLOT1:EGFP* was amplified form pSH1.6_Flot1:eGFP using primers CACC_OsFlot1-F1 and eGFPT2-R2, and cloned into pENTR following manufacturer's protocols.

Each fusion construct was cloned into *Agrobacterium* vector pIPKb001 (Himmelbach et al. 2007) using a Gateway LR Clonase II kit (Invitrogen) following the manufacturer's protocol. Both fusion constructs contained transcriptional and translational fusions, as verified by DNA sequencing. The plasmids for expressing *OsCLC1:EGFP* or *OsFLOT1:EGFP* driven by their native promoters were introduced into *A. tumefaciens* EHA105 using the freeze-thaw method (Holsters et al. 1978). Mature seed-derived callus from rice (*O. sativa Japonica* cv. YT16) was used for *Agrobacterium*-mediated transformation (Park et al. 2001). Following inoculation with *A. tumefaciens*, callus was transferred to regeneration medium and incubated in a growth chamber for 4 to 10 wk at 25˚C under a 16-h light photoperiod (General Electric Cool White 110 watts fluorescent bulbs, 40 to 50 µmol m^−2^ s^−1^). The regenerated shoots were transferred to rooting medium, incubated for 4 more weeks, and planted in Baccto Top Soil (Michigan Peat Co., Houston, Texas) for greenhouse propagation. Putative rice transformants were selected on 100 µg/ml hygromycin and expression checked by RT-qPCR and epifluorescence microscopy. Thirty independent T1 transgenic lines with CLC1:eGFP and 28 independent T1 lines with Flot1:eGFP were recovered. All lines were assayed by microscopy for fluorescence patterns and for fluorescence intensity. All lines for each construct showed the predicted fluorescence pattern. We chose lines with the highest levels of fluorescence for the assays.

### Silencing of rice genes

To evaluate the role of plant endocytosis on disease development and effector uptake, we performed VIGS using the Brome mosaic virus system developed in the R.S. Nelson laboratory (Noble Foundation, Ardmore, OK). We targeted OsAP2α, OsCHC1 and OsFLOT1 mRNA in rice cultivar IR64. The target rice sequences were cloned in the pC13/F3–13 m VIGS vector as described (Sun et al. 2013) and subsequently sequenced to verify sequence accuracy. Empty vector (pC13/F3-13m) was used as a negative control. pC13/F3-13m/AP-2α, pC13/F3-13m/CHC1, pC13/F3-13m/Flot1 or pC13/F3-13m (a control construct without an insert) were infiltrated with pC13/F1 + 2, after individual transformations of *Agrobacterium* using each plasmid, onto *Nicotiana benthamiana* plants and extract from inoculated leaves inoculated onto 7–10-day-old IR64 rice plants (all rice leaves were inoculated) as described by Wang et al. (2021), substituting rice plants for wheat plants. Rice plants were grown at 21 ± 2C temperature, with a day length of 16 h. Knockdown of the target genes was confirmed by RT-qPCR (Che Omar et al. 2016) at 15 d post inoculation. At this point, rice plants showing satisfactory reductions in transcript levels were used in our standard conidial spray inoculation and leaf sheath assays, except that the plant growth temperature was adjusted down to 21°C for the duration of the infection assay.

### Statistics

All experiments were performed with at least 3 biological replications, which are independent experiments with fungal cultures directly grown from frozen storage and different rice plantings. Biological replications included at least 2 technical repeats (independent assays with the same biological materials) to further confirm the reproducibility of the data. In all cases, technical and biological replications gave consistent results. Sample sizes, number of biological replicates, and the statistical tests used in each experiment are specified in the figure legends. Data were analyzed using an unpaired 2-tailed Student's *t*-test. *P* = 0.05 was considered nonsignificant, and exact values are shown where appropriate. All statistical analysis was performed using R Statistical Software (version 4.1.2) and Prism9 (GraphPad). Dot plots were routinely used to show individual data points and were generated using Prism9 (GraphPad). Bar graphs show the mean ± s.e.m. (unless otherwise stated) and were generated using Prism9 (GraphPad). Analysis of nonnormal datasets is represented by box-and-whisker plots that show the 25th and 75th percentiles, the median indicated by a horizontal line, and the minimum and maximum values indicated by the ends of the whiskers.

### Accession numbers

Sequence data for *M. oryzae* genes used in this study can be found in the GenBank/EMBL database under the following accession numbers: *BAS1*, FJ807764.1/NC_017844; *PWL1*, U36923.1; *PWL2*, U26313.1/NC_017853; *BAS4*, FJ807767.1/NC_017852.1; *BAS83*, MGG_08506.6/NC_017851.1; *BAS170*, MGG_07348.6/NC_017850.1. The rice genes used are as follows: *CLATHRIN LIGHT CHAIN 1* (*OsCLC1*), LOC4337419; *FLOTILLIN 1* (*OsFLOT1*), LOC4348926; *ADAPTOR PROTEIN COMPLEX 2α* (*OsAP2α*), LOC4331370; and *CLATHRIN HEAVY CHAIN 1* (*OsCHC1*), LOC4349546.

## Supplementary Material

koad094_Supplementary_Data

## Data Availability

All study data are included in the article and/or supporting information. All strains and plasmids generated in this study are available from the authors upon request.

## Supplemental data

The following materials are available in the online version of this article.

**Supplemental Figure S1.** Membranous effector compartments (MECs) are observed with additional fluorescent cytoplasmic effectors.

**Supplemental Figure S2.** MECs and host translocation co-occur during biotrophic invasion.

**Supplemental Figure S3.** Sequence similarities between Bas83 and Bas170.

**Supplemental Figure S4.** Bas83:mRFP identifies a separate membranous compartment surrounding BICs and BIC-associated IH cells.

**Supplemental Figure S5.** Strategy for targeted deletion of the *BAS83* gene.

**Supplemental Figure S6.** Rice OsCLC1:eGFP colocalizes with MECs labeled with additional fluorescent cytoplasmic effectors.

**Supplemental Figure S7.** VIGS silencing of endocytosis components in IR64 rice using the Brome Mosaic Virus system.

**Supplemental Table S1.** *M. oryzae* transformants used in this study.

**Supplemental Table S2.** Plasmids used in this study.

**Supplemental Table S3.** Oligonucleotides used in this study.

**Supplemental Movie 1.** Optical sections showing a BIC with MECs as well as translocated effector fluorescence in the rice cytoplasm.

**Supplemental Movie 2.** Optical sections showing MECs in the rice cytoplasm at a distance from a BIC in tissue stained by endocytosis tracker dye FM4-64.

**Supplemental Movie 3.** Fluorescently-labeled rice CLATHRIN LIGHT CHAIN 1 colocalizes with effector fluorescence in MECs.
