# Supplementary material for: Clathrin-mediated endocytosis facilitates the internalization of Magnaporthe oryzae effectors into rice cells
Source: Plant Cell. 2023 Mar 28;35(7):2527–51. doi: 10.1093/plcell/koad094 (PMC10291035; doi:10.1093/plcell/koad094)
Supplement: koad094_Supplementary_Data [file koad094_supplementary_data.zip › Legend for Supplemental Movies.docx]

Legend for Supplemental Movie 1. Optical sections showing a BIC with MECs as well as translocated effector fluorescence in the rice cytoplasm.

Legend for Supplemental Movie 2. Optical sections showing MECs in the rice cytoplasm and at a distance from a BIC in tissue stained by endocytosis tracker dye FM4-64.

Legend for Supplemental Movie 3. Fluorescently-labeled rice CLATHRIN LIGHT CHAIN 1 colocalizes with effector fluorescence in MECs.
