# Supplementary material for: Clathrin-mediated endocytosis facilitates the internalization of Magnaporthe oryzae effectors into rice cells
Source: Plant Cell. 2023 Mar 28;35(7):2527–51. doi: 10.1093/plcell/koad094 (PMC10291035; doi:10.1093/plcell/koad094)
Supplement: koad094_Supplementary_Data [file koad094_supplementary_data.zip › Supplemental_Figures_Tables-Oliveira-Garcia_etal-2023-Plant_Cell.pdf]

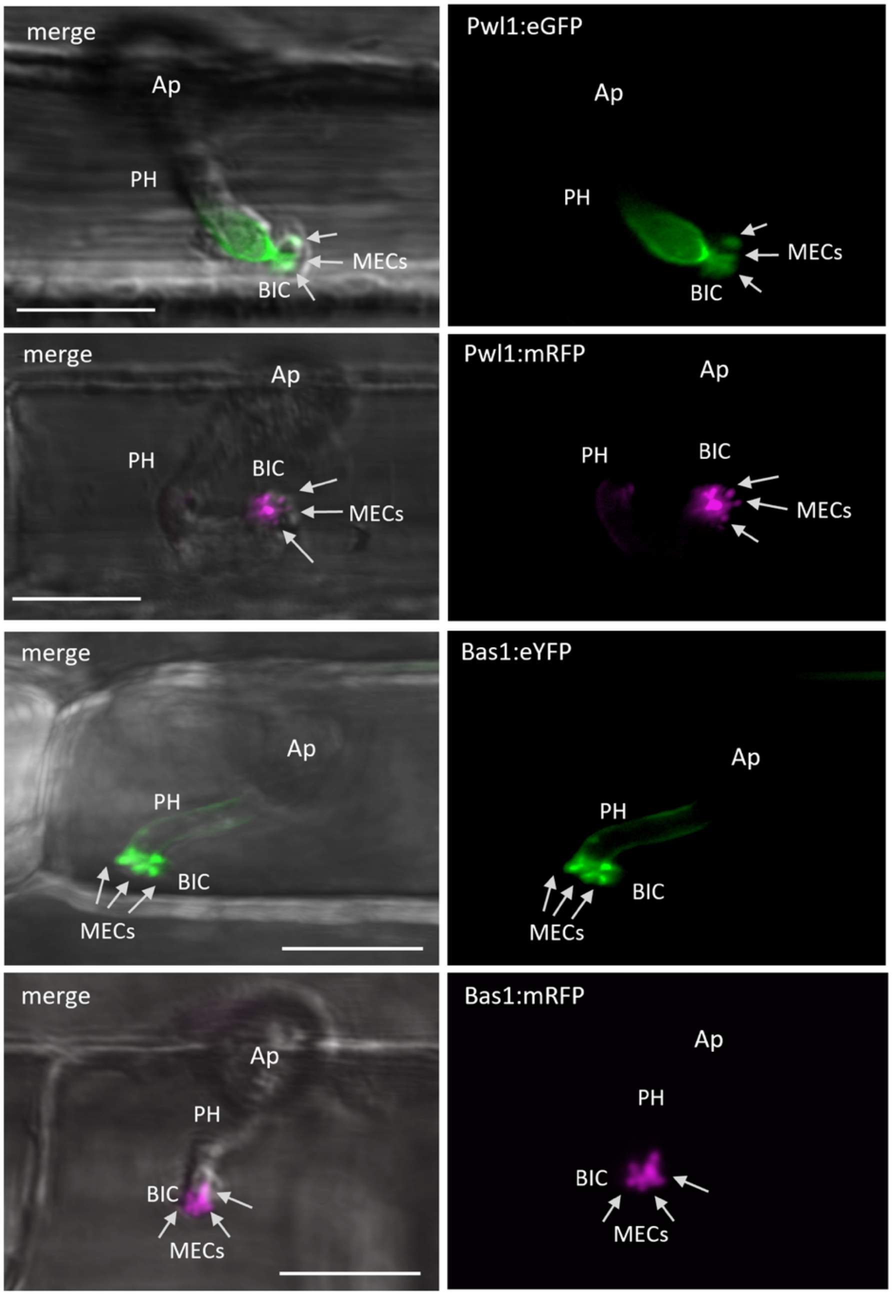

Supplemental Figure S1. Membranous effector compartments (MECs) are observed with additional fluorescent cytoplasmic effectors. (Supports Figure 1A-C)

MECs (upper to lower panels) are shown in BICs formed by strains KV174 and KV244 expressing Pwl1:eGFP (Pathogenicity towards weeping lovegrass 1) and Pwl1:mRFP, respectively, and by strains KV182 and KV170 expressing Bas1:eYFP and Bas1:mRFP, respectively. These results support Figure 1 by showing that MEC formation is independent of cytoplasmic effector and fluorescent protein used. BICs are imaged at the primary hyphal (PH) stage at ~19 hpi. Appressorial (Ap) penetration sites are observed in the merge images (left), which include bright field together with the fluorescence channel indicated on the right. Scale bars = 10 μm.

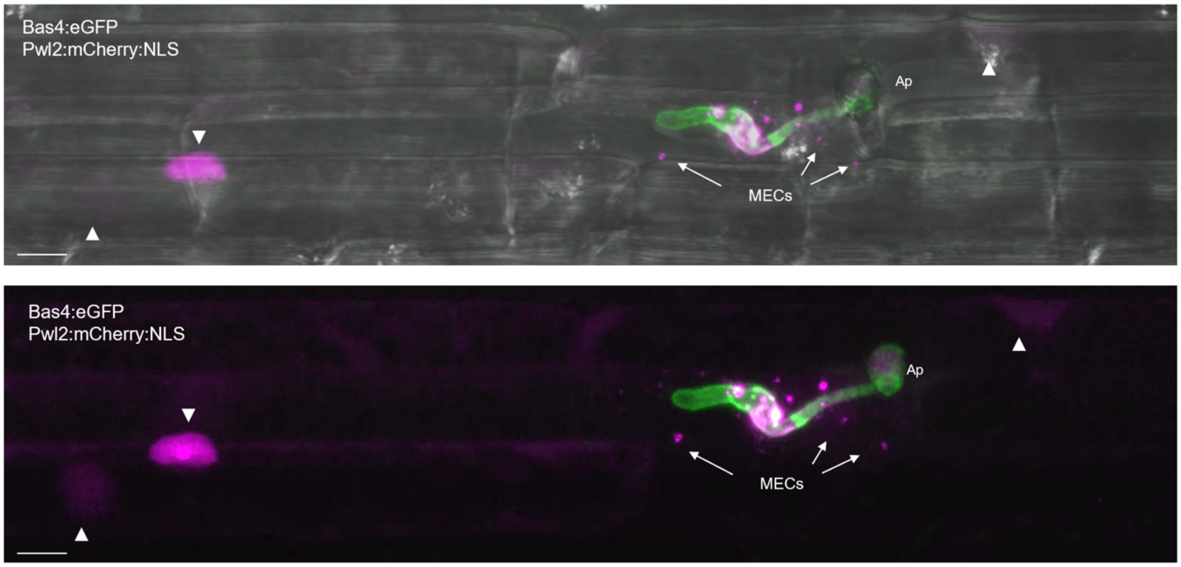

Supplemental Figure S2. MECs and host translocation co-occur during biotrophic invasion. (Supports Figure 1B)

Host translocation of Pwl2:mCherry:NLS (magenta) into the nucleus of the rice cell containing the bulbous IH and BIC in Figure 1B. This BIC, produced by strain KV168 expressing Pwl2:mCherry:NLS and Bas4:eGFP (green) at 24 hpi, occurs above the first bulbous IH cell. MECs (white arrows) are visible in the host cytoplasm at a distance from the BIC. Addition of an artificial nuclear localization signal (NLS) to the fluorescent Pwl2 fusion protein (Khang et al., 2010) facilitates visualization of effector translocation through accumulation in the host nucleus in both invaded and neighboring cells (white arrowheads). The Bas4:eGFP control shows that the EIHM still surrounds the IH. Images (top to bottom) are merged bright field, eGFP and mRFP fluorescence; and merged eGFP and mRFP. Scale bars = 10 µm.

A

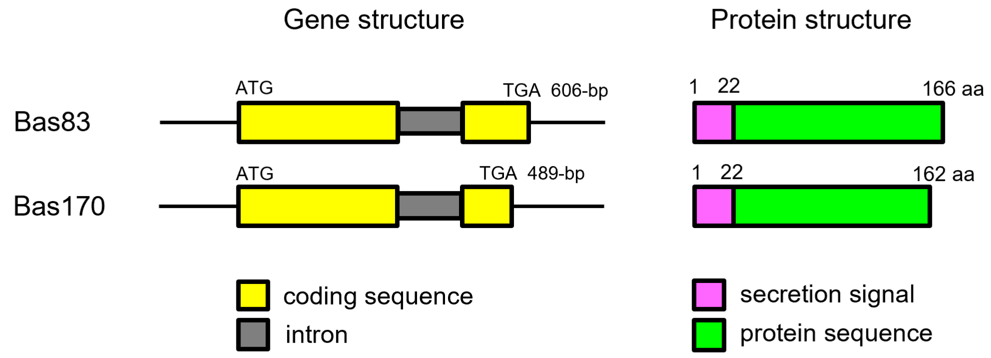

B

```
Bas83       MRFSTVFLPVVLAALQASGVSAGDESIGSTSPRRPGSPTDPGSLLAAGGSPGSRSAPA--   58
Bas170      MHFSKLLVPFALFVLNANGVQAASDGASTSGVKATGDMGSPRGVQDAGGSPGAPASSVAQ   60
            *:**.:::*..* .*:*.**.*..:. .::. :  *.  .* .:  ******: :: .

Bas83       -TDGALPPRDQGMIGFGPIPSARNRK-------------------KFPQPNIVPGPDGTA   98
Bas170      ASSGSSKPSRLTRISRIGRPKRSNRPNRPKSTKMCQTSLLSSTGVVVRTMSLVPGEDNLF   120
             :.*:  *     *.    *.  **                     .   .:*** *.

Bas83       AHPEGPMCIITLVRPFGRSESLPLRAGVEGIPDGWSWKWRKAPYAQDKKKSRVFGGLGRF   158
Bas170      SYPDGPDQTEHVITVKPSKLTCKPSITEGGIPEGWKWKYMKV------------------   162
            ::*:**     ::     . :        ***:**.**: *.

Bas83       GRTSQRTG     166
Bas170      --------     162
```

Supplemental Figure S3. Sequence similarities between Bas83 and Bas170. (Supports Figure 1D-F)

We identified cytoplasmic effector Bas170 (MGG_07348.6) in a search for homologs to the cytoplasmic effector gene Bas83 (MGG_08506.6), originally reported as encoding a biotrophy-associated secreted protein by Mosquera et al. (2009) and demonstrated to have a novel protein localization pattern in this study. **A.** Gene (left) and protein (right) structures of Bas83 and Bas170 in *M. oryzae* reference genome strain 70-15. Exon positions (yellow bars) are shown with one conserved intron (grey bars). The predicted proteins show a clear secretion signal peptide (magenta), but no conserved domains (green bars). **B.** Pairwise amino acid sequence alignment between Bas83 and Bas170 proteins shows low levels of amino acid sequence similarity.

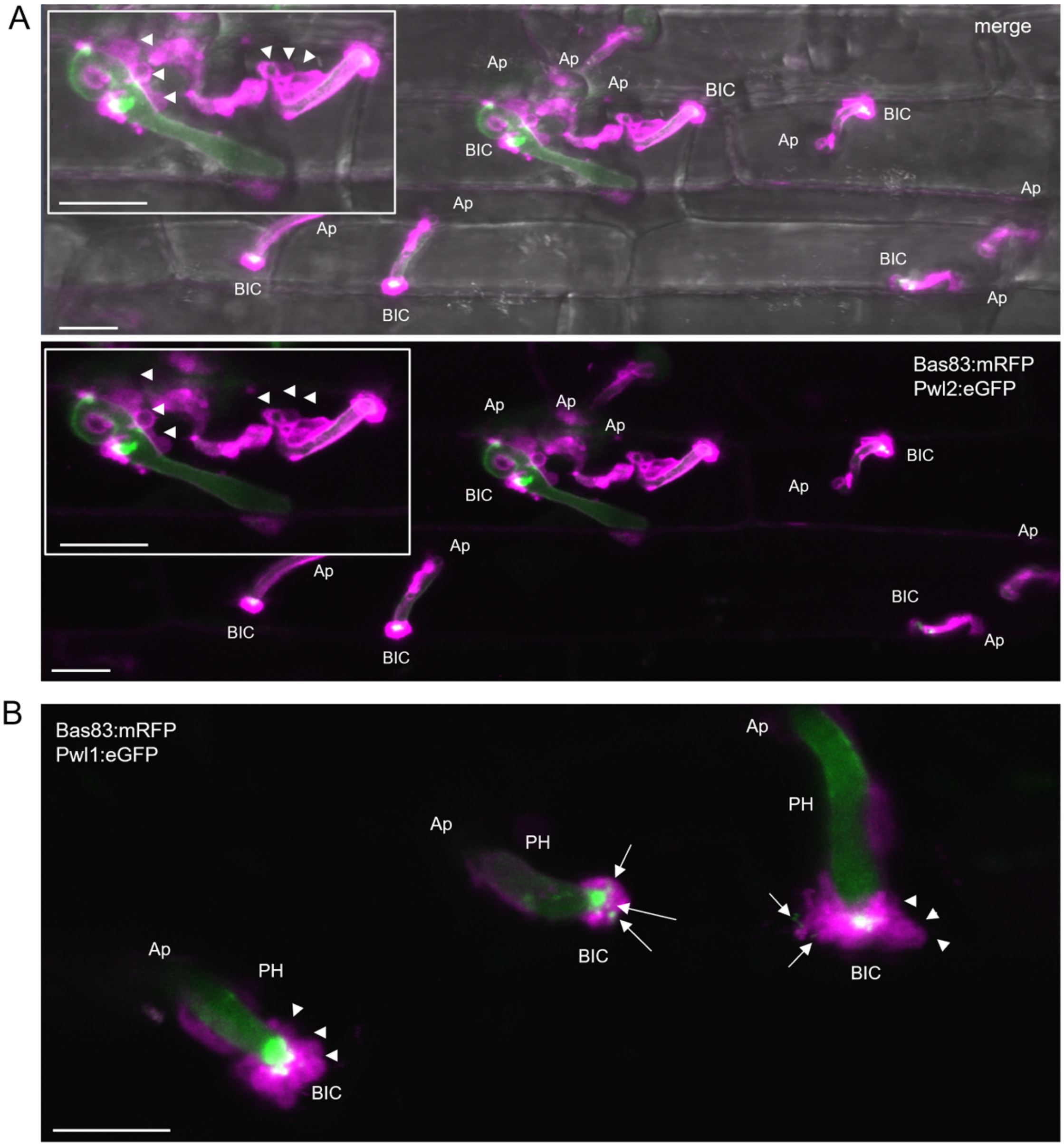

Supplemental Figure S4. Bas83:mRFP identifies a separate membranous compartment surrounding BICs and BIC-associated IH cells. (Supports Figure 4)

Multiple infection sites of strains KV222 and KV246 expressing respectively Bas83:mRFP with either Pwl2:eGFP or Pwl1:eGFP in YT16 rice at 22 hpi. **A**. Localization of Bas83:mRFP suggests manipulation of plasma membrane near appressoria (Ap), primary hyphae and BICs. Insert shows enlarged views of membranous compartments (arrowheads). **B.** Localization of Bas83:mRFP in membranous compartments that lack Pwl1:eGFP fluorescence and surround the BIC (arrowheads) and primary hyphae (PH). Arrows indicate MECs containing Pwl1:eGFP, perhaps being released from the BIC to rice cell cytoplasm. All images are projections of optical sections, and all show merged eGFP and mRFP fluorescence, with bright field also merged in the upper image in A. Scale bars = 10 μm.

A

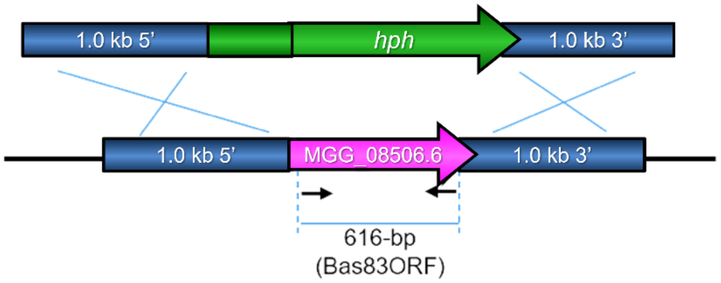

B

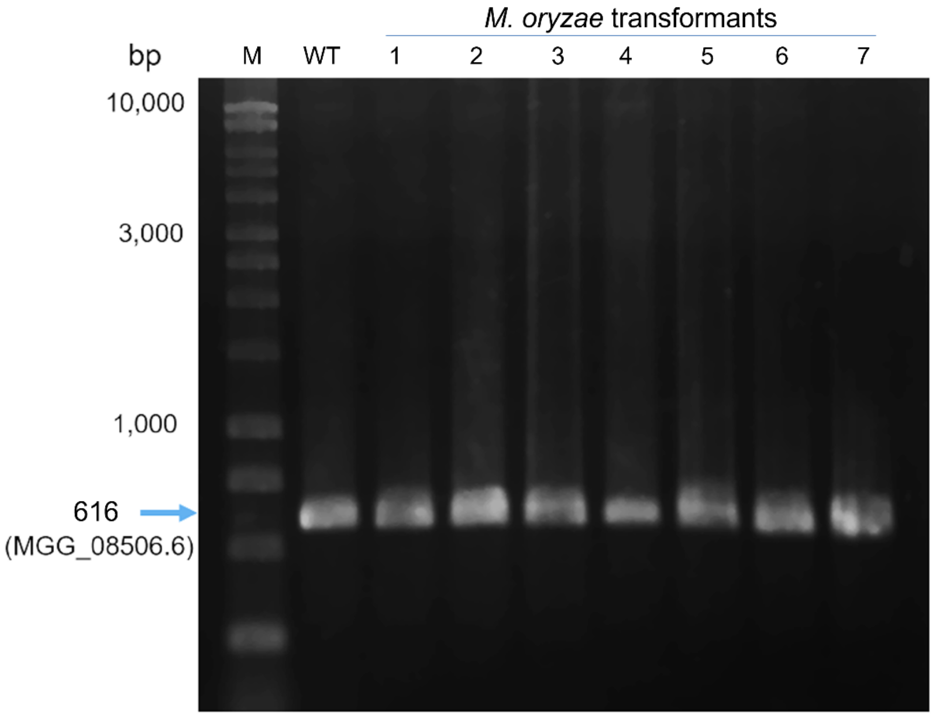

Supplemental Figure S5. Strategy for targeted deletion of the *BAS83* gene. (Supports Figure 4)

Both protoplast-based transformation and *Agrobacterium*-based transformation performed in two laboratories failed to generate knock-out mutations in *BAS83*. **A.** The deletion cassette consisted of the hygromycin cassette cloned between ~1.0 kb 5'- and 3'-flanking sequences of *BAS83*. Arrows indicate binding sites of primers Bas83:BASKOtest-F1 and Bas83:BASKOtest-R1; the bar indicates the predicted ~616 bp-PCR band in the wild-type (WT) strain and transformants with an ectopically integrated deletion cassette. Not to scale. **B.** PCR screen for homologous integration of the deletion cassette. PCR primers (Bas83:BASKOtest-F1 and Bas83:BASKOtest-R1) have binding sites in the coding sequence of *Bas83*, so that the 616 bp band can only be amplified from the wild-type (WT) strain and strains harboring an ectopically integrated deletion cassette. Seven independent transformants (1 – 7) of a total of 400 are shown. PCR analyses showed a ~616 bp band for all transformants tested, indicating ectopic integration of the deletion cassette. M indicates the DNA size marker.

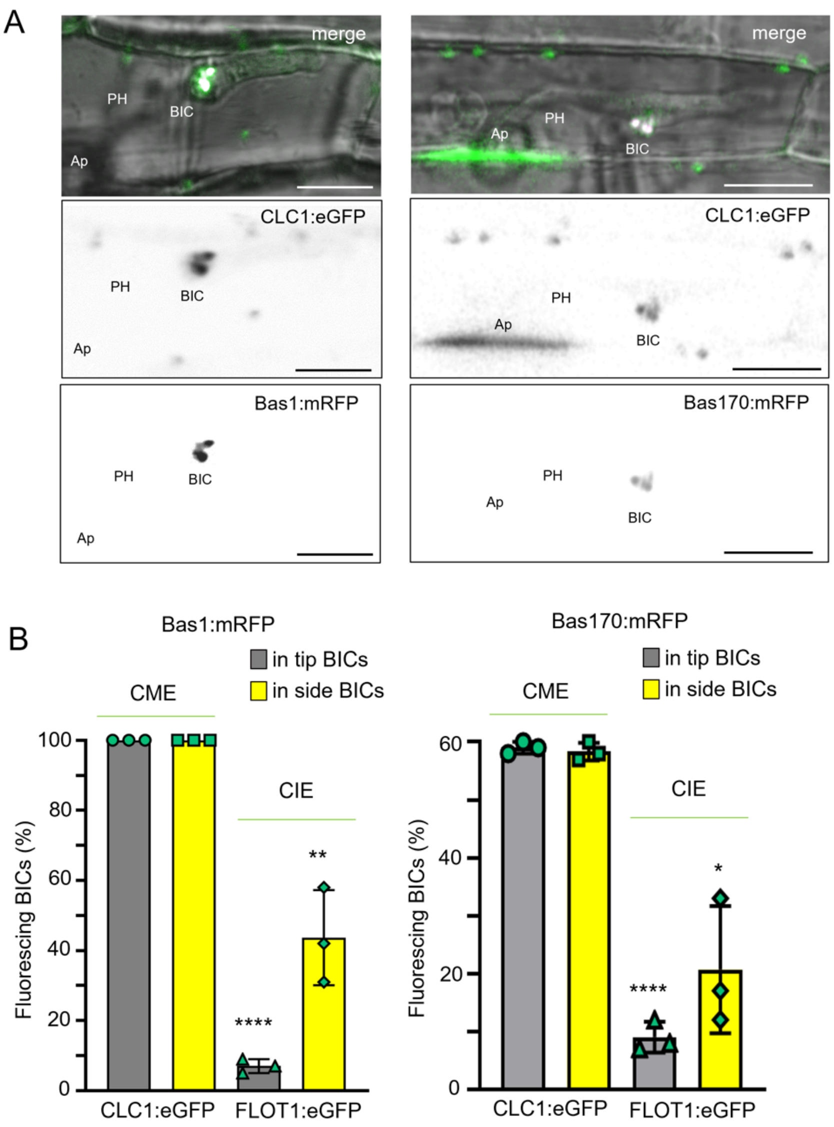

Supplemental Figure S6. Rice OsCLC1:eGFP colocalizes with MECs labeled with additional cytoplasmic effectors. **(**Supports Figure 6)

A. Rice leaf sheaths expressing OsCLC1:eGFP were inoculated with strain KV170 expressing Bas1:mRFP (left panels) or strain KV224 expressing Bas170:mRFP (right panels). These infected rice cells contain bulbous IH with side-BICs. Cell wall autofluorescence is seen below the appressorium in the image with Bas170:mRFP. Images shown top to bottom are merged bright field, eGFP (green) and mRFP (magenta); then eGFP alone and mRFP alone as black and white inverse images. Scale bars = 10µm. **B.** Quantification of colocalization of OsCLC1:eGFP or OsFlot1:eGFP with MECs labeled by Bas1:mRFP (left) or Bas170:mRFP (right) in tip- or side-BICs. Bars are standard deviations. ****P<0.0001, **P=0.002, *P=0.038; Bas1mRFP/CLC1:eGFP colocalization: three times 98 BICs observed; Bas170mRFP/CLC1:eGFP colocalization: three times 60 BICs observed.

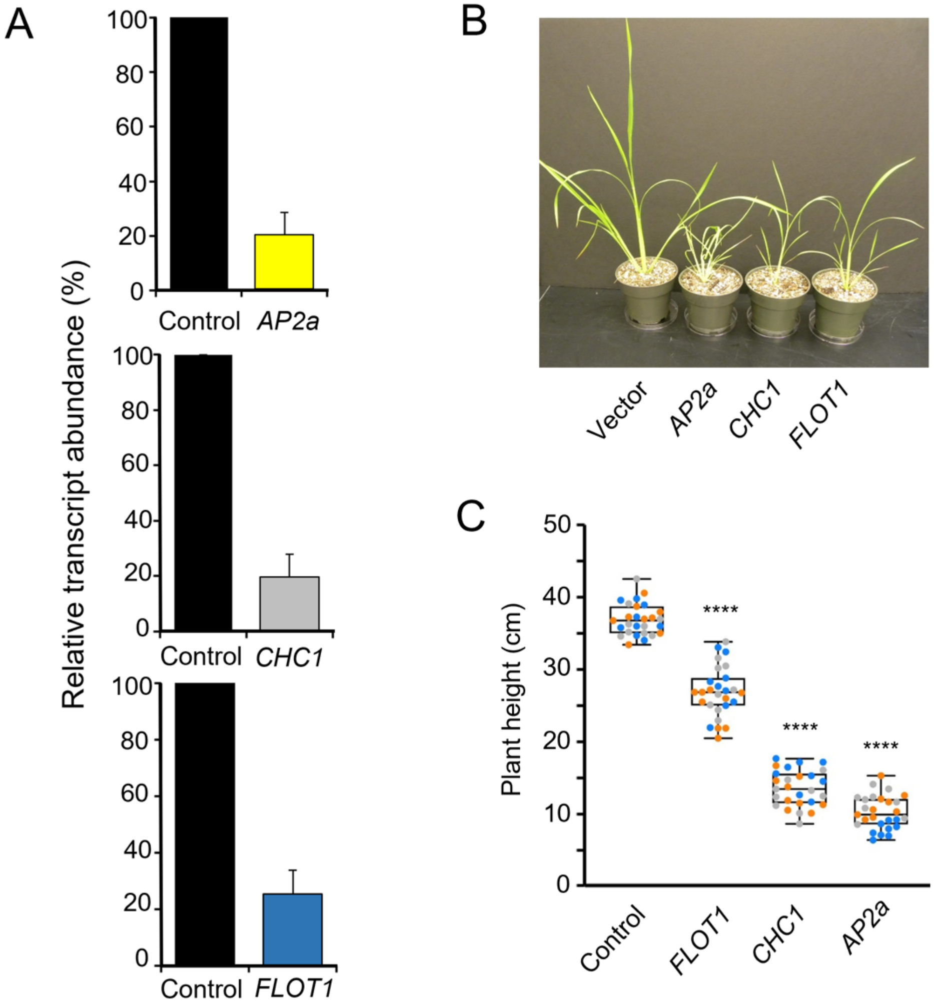

Supplemental Figure S7. VIGS silencing of endocytosis components in IR64 rice using the Brome Mosaic Virus system. (Supports Figure 7)
A. Relative transcript abundance of rice *OsAP2*α, *OsCHC1*, and *OsFlot1* in lines expressing RNAi*AP-2*α, RNAi*CHC1*, RNAi*Flot1* silencing constructs relative to vector control plants at 15 days post inoculation. Individual plants showing high levels of silencing were chosen for infection assays. Three biological replications with 9 plants each were evaluated. Error bars indicate standard deviations. **B.** *OsAP2*α, *OsCHC1* and *OsFlot1* silenced plants show significant stunting compared to plants that received the vector control, as expected for inhibiting critical gene functions. **C.** Quantification of plant height for silenced plants and the vector control using box-and-whisker plots with individual data points (blue, gray and orange data points represent three biological replicates of nine plants each); the boxes show the 25th and 75th percentiles, the median is indicated by a horizontal line and the minimum and maximum values by the ends of the whiskers. ****$P<0.0001$.

Supplemental Table S1: Transformants used in this study

Strain	Used In:	Effector:FP	Description (background strain; plasmid)
KV168	Fig.1B; Sup. Fig. S2	Pwl2:mCherry:NLS & Bas4:eGFP	Guy11; pBV591 (Hyg^R)
KV170	Fig. 2B; Sup. Fig. S1; Sup. Fig. S6	Bas1:mRFP	Guy11; pBV440 (Gen^R)
KV174	Sup. Fig. S1	Pwl1:eGFP	Guy11; pBV249 (Hyg^R)
KV176	Fig. 3D,E; Fig. 5C; Sup. Movie S2	Pwl2:eGFP	Guy11; pBV252 (Hyg^R)
KV182	Sup. Fig. S1	Bas1:eYFP	Guy11; pBV231 (Hyg^R)
KV209	Fig. 3A-C; Fig. 4A-D; Fig. 5A,B; Fig. 6A-D; Fig. 7D-F; Fig. 9A-E; Sup. Movie S3	Pwl2:mRFP	Guy11; pBV1192 (Gen^R)
KV211	Fig. 2A-F; Sup. Movie S1	Pwl2:mRFP and Bas1:eYFP	Guy11, pBV231 (Hyg^R); pBV1192 (Gen^R)
KV217	Fig. 1A,C; Fig. 7C; Fig. 8A-C	Pwl2:mRFP & Bas4:eGFP	Guy11, pBV436 (Hyg^R)
KV220	Fig. 4B,C	Bas83:mRFP	Guy11, pBV1194 (Gen^R)
KV222	Fig. 4A; Sup. Fig. S4A	Bas83:mRFP & Pwl2:eGFP	Guy11, pBV252 (Hyg^R); pBV1194 (Gen^R)
KV224	Fig. 1D-F; Sup. Fig. 2; Sup. Fig. S6	Bas170:mRFP	Guy11, pBV1196 (Gen^R)
KV244	Sup. Fig. 1	Pwl1:mRFP	Guy11, pBV1211 (Gen^R)
KV246	Sup. Fig. S4B	Bas83:mRFP & Pwl1:eGFP	Guy11; pBV249 (Hyg^R); pBV1194 (Gen^R)

Supplemental Table 2: Key plasmids used in this study

Clone	Description
pBV231	*BAS1* (MGG_04795.6) promoter and entire coding sequence with a C-terminal translational fusion of the eYFP reporter gene. A 1.3-kb PCR product containing the *BAS1* gene was amplified by PCR, digested with *Eco*RI and *Bam*HI, and subsequently cloned in *Eco*RI-*Bam*HI sites of pBV181 containing eYFP in pBHt[2] (AddGene Plasmid #104175). (Hygromycin^R, Kanamycin^R) Published in Mosquera et al. (2009). *Used in transformants KV182 and KV211.*
pBV249/ pSK1898	*PWL1* (GenBank:U36923.1) promoter and entire coding sequence with a C-terminal translational fusion of the eGFP reporter gene. A 1,171-bp *Kpn*I-*Bam*HI fragment of pSK1876 (P_{PWL1}:PWL1CDS) and the 986-bp *Bam*HI-*Xba*I fragment of pSK1873 (eGFP:Ter) cloned between the *Kpn*I and *Xba*I sites of pBHt2. Plasmid published in Khang et al. (2010). *Used in KV174 and KV246.*
pBV252/ pSK1901	*PWL2* (MGG_04301.6) promoter and entire coding sequence with a C-terminal translational fusion of the eGFP reporter gene. Plasmid published in Khang et al. (2010). *Used in transformants KV176 and KV222.*
pBV367	mRFP binary expression vector derived from pBGt (Seogchan Kang, Pennsylvania State University), consisting of three modules: P27 promoter (*Eco*RI-*Bam*HI fragment), mRFP (*Bam*HI-*Sph*I fragment), and *N. crassa* β-tubulin terminator (*Sph*I-*Hind*III fragment) cloned in *Eco*RI-*Hind*III sites of pBGt. (Geneticin^R, Kanamycin^R) Plasmid published in Giraldo et al. (2013).
pBV436	Plasmid expressing P_{PWL2}*:PWL2CDS:mRFP:Ter* and P_{BAS4}*:BAS4CDS:eGFP:Ter* cloned in the *Eco*RI and *HindIII* sites of pBHt2 (AddGene #104175). Published in Khang et al (2010). *Used in KV217.*
pBV440	*BAS1* (MGG_04795.6) promoter and entire coding sequence with a C-terminal translational fusion of the mRFP reporter gene. Plasmid published in Mosquera et al. (2009). *Used in transformant KV170*.
pBV591	Used to express Pwl2:mCherry:NLS (with an added nuclear localization signal) together with Bas4:eGFP. ProPWL2:PWL2CDS:mCherry:NLS:Tnos and ProBAS4:BAS4CDS:eGFP:Ter cloned in *Eco*RI and *Hind*III sites of pBHt2. (Hygromycin^R). Published in Khang et al. (2010). *Used in KV168.*
pBV1192	*PWL2* (MGG_04301.6) promoter and entire coding sequence with a C-terminal translational fusion of the mRFP reporter gene. A 1.7-kb *Pwl2* gene fragment cloned into *EcoR*I-*BamH*I restriction sites of pBV367 (Geneticin^R, Kanamycin^R). *Used in transformants KV209 and KV211.*
pBV1194	*BAS83* (MGG_08506.6) promoter and entire coding sequence with a C-terminal translational fusion of the mRFP reporter gene. A 1.6-kb *BAS83* gene fragment cloned into *EcoR*I-*BamH*I restriction sites of pBV367. (Geneticin^R, Kanamycin^R). *Used in transformants KV220, KV222 and KV246.*

pBV1196	*BAS170* (MGG_07348.6) promoter and entire coding sequence with a C-terminal translational fusion of the mRFP reporter gene. A 2.0-kb *BAS170* gene fragment cloned into the *EcoR*I restriction site of pBV1214. (Geneticin^R, Kanamycin^R). *Used in transformant KV224.*
pBV1198	Clathrin-mediated endocytosis (CME) fluorescent component for expression in rice. A 4.71-kb fragment of *CLATHRIN LIGHT CHAIN 1* (Os*CLC1*) (promoter and entire ORF of rice *OsCLC1* gene; LOC4337419) integrated into *Kpn*I and *Xho*I restriction sites of pSH1.6_EGFP (AddGene #42323). A 5.7-kb CLC1:eGFP construct was PCR amplified and integrated into pENTR (pENTR™/D-TOPO™ Cloning Kit, ThermoFisher Scientific) and transferred to *Agrobacterium* vector pIPKb001 (Himmelbach et al., 2007) using the Gateway LR Clonase II system. *Used to express OsCLC1:eGFP in rice cv. YT16.*
pBV1200	Clathrin-independent endocytosis (CIE) - associated fluorescent fusion protein for expression in rice. A 4.4-kb fragment of *FLOTILLIN 1* (promoter and entire ORF of rice *OsFLOT1* gene; LOC4348926) integrated into *Hind*III and *Eco*47III restriction sites of pSH1.6_EGFP (AddGene #42323). A 5.4-kb OsFLOT1:eGFP construct was PCR amplified and integrated into pENTR (pENTR™/D-TOPO™ Cloning Kit, ThermoFisher Scientific) and transferred to *Agrobacterium* vector pIPKb001 (Himmelbach et al., 2007) using the Gateway LR Clonase II system. *Used to express OsFLOT1:eGFP in rice cv. YT16.*
pBV1202	VIGS vector for silencing the *OsFLOT1* gene in rice cv. IR64. A 0.3-kb fragment for silencing of the rice gene for CIE component FLOTILLIN 1 (*FLOT1* gene; LOC4348926) was cloned in *Avr*II-*Nco*I restriction sites of the pC13/F3-13m VIGS vector (Kanamycin^R) as described in Wang et al. (2021).
pBV1204	VIGS vector for silencing the *AP-2α* gene in rice. A 0.3-kb fragment for targeted silencing of rice gene for CME component *ADAPTER PROTEIN COMPLEX 2α* (Os*AP2α* gene; LOC4331370) was cloned into *Avr*II-*Nco*I restriction sites of the pC13/F3-13m VIGS vector (Kanamycin^R) (Wang et al., 2021).
pBV1206	VIGS vector for silencing the *OsCHC1* gene in rice cv. IR64. A 0.3-kb fragment for targeted silencing of the rice gene for CME component *CLATHRIN HEAVY CHAIN 1* (*OsCHC1* gene; LOC4349546) was cloned in *Avr*II-*Nco*I restriction sites of the pC13/F3-13m VIGS vector (Kanamycin^R) (Wang et al., 2021).
pBV1211	*PWL1* (GenBank:U36923.1) promoter and entire coding sequence with a C-terminal translational fusion of the *mRFP* reporter gene. A 1.5-kb *PWL1* gene fragment cloned into the *Bam*HI restriction site of pBV367. (Geneticin^R, Kanamycin^R). *Used in transformant KV244.*
pBV1214	mRFP binary expression vector derived from pBGt (Seogchan Kang, Pennsylvania State University), consisting of four modules: P27 promoter (*Eco*RI-*Bam*HI fragment), multiple cloning site from pSH1.6_EGFP containing an extra *Eco*RI site cloned into the *Bam*HI restriction site, mRFP (*Bam*HI-*Sph*I fragment), and *N. crassa* β-tubulin terminator (*Sph*I-*Hind*III fragment) cloned in *Eco*RI-*Hind*III sites of pBGt. (Geneticin^R, Kanamycin^R).

Supplemental Table 3: Oligonucleotides used in this study

Primer name	Sequence 5' -3'	Description
**Primers used to fluorescently label effectors in *M. oryzae***		
MoPLW2-EcoRI-F1*	AAAGAATTCTTACCCGTGGCAAGGATAAC	1.6-kb *Pwl2* promoter plus entire coding sequence
MoPLW2-BamHI-R1	TTTGGATCCCATAATATTGCAGCCCTCTTCTCG	
MoPLW1-BanHI-F1	AATGGATCCGCCTGTCACTAGGGCTTCTG	1.5-kb *Pwl1* promoter plus entire coding sequence
MoPLW1-BamHI-R1	ATTGGATCCCATAATTGGCAGCCCTGATCTC	
mRFPORF1-R1	CTCCTCGCCCTTGCTCACCAT	1.5-kb test PCR to check the orientation of *PWL1* in pBV367
MoBas83EcoRI-F1	ATAGAATTCAGAGTGTGCGCTAGCTCGAC	2.0-kb *Bas83* promoter plus entire coding sequence
MoBas83BamHI-R1	TATGGATCCACCCGTCCGCTGCGAAGTC	
MoBas170EcoRI-F1	AAAGAATTCTCGTTGCAGCGGCAGTCAAG	2.0-kb *Bas170* promoter plus entire coding sequence
MoBas170EcoRI-R1	AAAAGAATTCGCCGTCTCCACAGCCCTGGAT	
MoBas170ORF-F1	GTGTCGTGGTTAGGACAATG	0.772-kb test PCR to check the orientation of *Bas170* in pBV367; used with the primer mRFPORF1-R1
**Primers used to gene replacement in *M. oryzae***		
M13F	CGCCAGGGTTTTCCCAGTCACGAC	1.113-kb of HY split cassette (Catlett et al., 2003)
HYsplit	GGATGCCTCCGCTCGAAGTA	
M13R	AGCGGATAACAATTTCACACAGGA	0.74-kb of YG split cassette (Catlett et al., 2003)
YGsplit	CGTTGCAAGACCTGCCTGAA	
Bas83:BASKOtest-F1	ATGCGATTCTCGACCGTTTTC	0.603-kb of the *Bas83* ORF (test PCR to verify the presence of *Bas83* gene in the transformants
Bas83:BASKOtest-R1	ACCCGTCCGCTGCGAAGTC	
Bas83LF-F	AGAGTGTGCGCTAGCTCGAC	1.0-kb 5'-flanking region of MGG_08506.6; used with the split marker method
Bas83LFHY-R	*GTCGTGACTGGGAAAACCCTGGCG*GCAAGGCGGCCAGGACAAC	
Bas83RFYG-F	*TCCTGTGTGAAATTGTTATCCGCT*ACGCGTCGTTGTAGTGACTTG	

Bas83RF-R	TAACAGCAGTCTGCCCAACAC	1.0-kb 3'-flanking region of MGG_08506.6; used with the split marker method
Bas83LFHYG-R	*CCTCCACTAGCTCCAGCCAAGCC*GCAAGGCGGCCAGGACAAC	1.0-kb 5'-flanking region of MGG_08506.6; used with the primer Bas83LF-F
Bas83LFHYG-F	*TAGAGTAGATGCCGACCGCGGGTT*ACGCGTCGTTGTAGTGACTTG	1.0-kb 5'-flanking region of MGG_08506.6; used with the primer Bas83RF-R
HYG-1F	GGCTTGGCTGGAGCTAGTGGAGG	1.4-kb hygromycin gene
HYG-2R	AACCCGCGGTCGGCATCTACTCTA	
**Primers used to silence endocytic machinery in rice**		
OsFlot1AvrII-F1	AAACCTAGGGTGTATATTGCTGTGTAACTG	0.3-kb *Flot1* RNAi sequence target
OsFlot1NcoI-R1	TTTCCATGGTGCAGTTATATACTCATGTAAATC	
OsClat1HeavyAvrII-F1	AAACCTAGGAGGAGGCATCTTTCAAGTTGTAG	0.3-kb *CHC1* RNAi sequence target
OsClat1HeavyNcoI-R1	TTTCCATGGGACATGAGACCGTAAACAAGTAAC	
OsAP-2AvrII-F1	AAACCTAGGGCGGAGCTTTCTTTCTAG	0.3-kb *AP-2α* RNAi sequence target
OsAP-2NcoI-R1;	TTTCCATGGGAATTCACCCATCAATTTAATAAG	
OsFlotillin1-ORF-F1	ATCAACGCCGACGCCATCAG	qRT-PCR primers for rice *Flot1*
OsFlotillin1-ORF-R1	GGCGGCAGCATCTTGTACAC	
OsAP2a-ORF-F1	GAACAGGCCGCCAATTCAG	qRT-PCR primers for rice *AP-2α*
OsAP2a-ORF-R2	TGATCCAGCCCTTGTGATGTAG	
OsCHC1-ORF-F1	ATGCCCAATTGCTTCCTCTC	qRT-PCR primers for rice *CHC1*
OsCHC1-ORF-R2	ATCCCATATGCTGGCATAGG	
EF F2	CCGCCAAGAAGAAATGAGCA	qRT-PCR primers for rice *EF-1α* gene (Ding et al., 2006)
EF R2	TCCATGCAACGAGTGCCAT	
**Primers used to fluorescently label endocytosis markers in rice**		
KpnI_OsCLC1Prom1-F1	TTGGTACCCTGGAGAGCCCAGGCATTAC	4.71-kb Promoter and entire ORF of rice *CLC1* gene
XhoI_OsCLC1-R1	CGCTCGAGGCTCCGATGCTGCAGGCTGCTC	

CACC_OsCLC1-F1	CACCCTGGAGAGCCCAGGCATTAC	5.657-kb CLC1:eGFP
eGFPT2-R2	TGAAGGCGTACTAGGTTGCAGTC	
HindIII-OsFlot1Prom-F1	AAAAGCTTCAAAGCCGCTAACGGTTTGG	4.424-kb Promoter and entire ORF of rice *Flot1* gene
Eco47III_OsFlot-R1	AAAAGCGCTGGACTGGTCGACGAGGGGGCGC	
CACC_OsFlot1Prom1-F1	CACCCAAAGCCGCTAACGGTTTGG	5.367-kb Flo1:eGFP; used with the primer eGFPT2-R2

*Restriction sites are underlined
