# Supplementary material for: Clathrin-mediated endocytosis facilitates the internalization of Magnaporthe oryzae effectors into rice cells
Source: Plant Cell. 2023 Mar 28;35(7):2527–51. doi: 10.1093/plcell/koad094 (PMC10291035; doi:10.1093/plcell/koad094)
Supplement: koad094_Supplementary_Data [file tpc22.00359.PeerReviewReport.pdf › tpc22.00359.PeerReviewReport.pdf]

Clathrin-mediated endocytosis facilitates the internalization of *Magnaporthe oryzae* effectors into rice cells

Ely Oliveira-Garcia, Tej Man Tamang, Jungeun Park, Melinda Dalby, Magdalena Martin-Urdiroz, Clara Rodriguez Herrero, An Hong Vu, Sunghun Park, Nicholas J. Talbot & Barbara Valent

Corresponding authors: E. Oliveira-Garcia, EOliveiraGarcia@agcenter.lsu.edu and B. Valent, E-mail: bvalent@ksu.edu.

Review timeline:

**TPC2022-RA-00187**	Submission received:	Feb. 17, 2022
	1st Decision:	April 4, 2022 *manuscript declined*
**TPC2022-RA-00359**	Submission received:	Dec. 5, 2022
	1st Decision:	Jan. 20, 2023 *revision requested*
**TPC2022-RA-00359R1**	1st Revision received:	Feb. 23, 2023
	2nd Decision:	Mar. 2 2023 *acceptance pending, sent to science editor*
	Final acceptance:	Mar. 20, 2023

REPORT: (The report shows the major requests for revision and author responses. Minor comments for revision and miscellaneous correspondence are not included. The original format may not be reflected in this compilation, but the reviewer comments and author responses are not edited, except to correct minor typographical or spelling errors that could be a source of ambiguity.)

TPC2022-RA-00187 1st Editorial decision – *declined* **April 4, 2022**

Thank you for choosing to send your manuscript entitled "Clathrin-mediated Endocytosis Facilitates Internalization of Magnaporthe oryzae Effectors into Rice Cells" for consideration at The Plant Cell. Your submission has been evaluated by members of the editorial board as well as expert reviewers in your field, and we regret to inform you that we are not able to recommend publication of this manuscript in its current form.

Reviewer 1 and Reviewer 2 both agree that this is a solid piece of work and may contribute to our understanding of fungal effector secretion into plant cells. However, they also brought up some critical concerns, such as the lack of detailed experimental and analysis methods (especially the measurement of vesicle sizes, which are inconsistent with CCVs, and some are even beyond the detection limit of the light microscope). They also suggested further validation and clarification of some key results using alternative methods (e.g. the use of alternative CME/endocytosis inhibitors). Reviewer 3 also recognized the conceptual importance of this work but is less convinced by the data shown in the current format. They are particularly concerned with the "identity" of observed vesicles and potential bleed through in some fluorescence imaging data.

During the post-review consultation session, we agreed that if you could address the major points raised by the reviewers by new experiments, we would welcome a resubmission. This may be treated as a new submission, but we would attempt to use at least some of the same reviewers. Nevertheless, reviewers will be asked to assess as a new manuscript (i.e. are the claims fully supported by the data and do the results presented move the field forward?), and not only whether previous reviewer comments have been addressed.

-- Reviewer comments:

[Reviewer comments shown below along with author responses]

TPC2022-RA-00359 Submission received **Dec. 5, 2022**

Reviewer comments on previously declined manuscript and **author responses:**

<u>Decision letter:</u>

> **We are happy that Reviewers 1 and 2 agree that this manuscript presents "a solid piece of work and may contribute to our understanding of fungal effector secretion into plant cells." We have addressed critical concerns by these reviewers. Specifically, we have discussed our experimental and analysis methods in greater detail as needed. We remeasured all vesicle sizes using ImageJ instead of our microscope software and the smallest vesicles are within the detection limits of our light microscope. In our detailed comments to reviewers, we discuss why we do not believe that it is a problem that our effector vesicles are larger than reported for CCVs in other studies. We document that the**

effector vesicles are increasing in size over time, as well as decreasing in numbers as the fungus grows to fill the host cell. This vesicle-size and abundance dynamics suggests pathogen-influenced factors impact the effector internalization process as host cell invasion progresses. It was a good idea to test new clathrin heavy chain-targeted inhibitors Endosidin 9, and especially the less toxic derivative, Endosidin9-17. New data added using these inhibitors fully supports our previous conclusions. We are also happy that Reviewer 3 "recognized the conceptual importance of this work." Although this reviewer said that our "co-localization results are convincing," the reviewer rightly pointed out that "CLC1-eGFP should also be detected at the vesicles but no vesicles were shown". We had included one example of CLC1-eGFP colocalization with effector vesicles in Supplemental movie S3, but this was easy to miss. We have now added new images and supporting data that clearly show CLC1-eGFP co-localizes with effector vesicles, and not just with the broader BIC region. We discuss controls and complementary experiments with CIE markers that rule out bleed through in our fluorescence imaging data. We are very happy that our figures have been greatly improved through the process of converting them to magenta and green as requested. Additional point-by-point responses are included in our detailed response to reviewers.

It will be important to convince the editors and reviewers that the study adds significant new understanding(s) of mechanisms or processes, or otherwise represents a significant advance in the field, and that the major claims made are fully justified by the data presented. This includes careful consideration and explanation of the various controls used in experiments, the extent and manner of replication, and the statistical analyses used. Sampling methods and the nature of "biological replicates" should be described precisely (i.e. different plants, parts of plants, pooled tissue, independent pools of tissue, sampled at different times, etc.), along with a clear description of and rationale for any statistical analyses conducted. The reader should know exactly what was sampled; what forms the basis of the calculation of any means and other statistical variables and parameters reported. This is also necessary to ensure that proper statistical analysis was conducted.

We have worked on clarifying the nature of our biological replicates, our experimental methods, especially measuring vesicle sizes, and our use of statistics. We apologize for the limited detail provided initially.

Reviewer #1:

The manuscript by Oliveira-Garcia et al set out to determine how fungal pathogens enter plants to cause infection, which is a significant question in plant biology. They use a combination of genetic and pharmacological agents to determine this entry is mediated by clathrin-mediated endocytosis (CME) as opposed to a clathrin independent pathway. In doing so, they also identify a novel cytoplasmic effectors and differences in the vesicles they are localized in. The authors should be praised for a full and fair discussion section.

We thank you for these positive comments.

Overall, the paper is well constructed and proceeds with a logical progression. The novelty lies in the fact that the authors identify a specific pathogen which internalizes via CME as even the authors note in the introduction, CME has been well established as the major mechanism of internalization. It is it however great to see that the flotillin CIE pathway is ruled out in this process.

Thank you for your supportive comments. However, we don't agree that "CME has been well established as the major mechanism of internalization." There is, to our knowledge, no direct evidence presented that effectors are delivered into plant cells by CME. There are a few previous reports for individual effectors, at least one implicating CIE, which are far from satisfactory as an explanation of fungal effector delivery. Our motivation for this study was to investigate the delivery mechanism in detail to provide new insight into effector delivery in *M. oryzae.* Our work provides the first data to implicate CME as a general effector internalization mechanism, using multiple distinct effectors, in blast disease. Our view is that earlier published studies are not close to establishing a general internalization mechanism.

We do see some minor impact from inhibiting CIE. Therefore, our results have not completely ruled out any role for CIE in blast effector accumulation, although it appears much less important than CME. Flot1 localization, VIGS and chemical inhibition of CIE indicates there could be a minor role of the rice CIE machinery for blast effector internalization. We have made every effort to present our results in a clear and dispassionate manner, highlighting limitations to the study and being measured in our interpretations. We believe this is essential in such studies, especially given previous studies of RXLR effector delivery mechanisms, which have not turned out to be robust.

Further novelty is in the finding of a novel cytoplasmic effector, but is not followed up or elaborated on.

We have now added more detail regarding two novel effectors, Bas170 and Bas83, which we feel addresses this comment and strengthens the novelty of our study further.

My major concern with the role of actin presented in their model. While the authors show that there is co-localization between actin and BICs, there is no evidence of a functional interaction presented. This point is critical as the role of actin in plant CME appears to be controversial (even noted by the authors in their discussion). In contrast to yeast, evidence from Arabidopsis and Chara show that actin is not required for CME (Narasimhan et al 2020 and Klima & Foissner 2008). Therefore, for the authors to place actin at such a prominent position in their model (i.e. at the neck driving formation of the vesicle), extensive further work is required to make such a bold claim. For example, high resolution imaging of CME events with actin inhibition and/or markers.

Thank you for this comment. We agree that our model was potentially misleading as it may have over-stated the importance of actin in effector internalization. We have therefore changed the model accordingly. We do believe, however, that our results showing the localization of actin at BICs are important for the study, demonstrating the cytoskeletal organization around these membrane-rich structures that are involved in effector delivery. We have made clear acknowledgement that the exact role of actin in CME is unknown.

It is not clear to me how the authors were able to resolve the size of vesicles to values below the diffraction limit using standard confocal microscopy. The resolution limit is physically limited to 200 nm, but the authors report sizes well below this (sometime down to 41 nm).

Thank you for pointing out this problem. The reviewer is correct. Our original size estimates were carried out with our microscope software, which is clearly inaccurate. We have now re-calculated all vesicle measurements using ImageJ, and now present realistic numbers for vesicle size. We have explained in detail how we made the measurements so others can repeat experiments independently to validate the vesicle sizes reported. Methods for measurement of vesicle sizes by confocal microscopy have limitations. Even if our measured sizes are not precise, the changes in vesicle sizes and numbers through time is a robust result, and provides a clue for further mechanistic studies. We believe precise vesicle sizes are not essential data for this publication.

The manuscript would greatly benefit with the addition of further explanations about methods and analysis. Critically, there is no details in the methods explaining how these analyses were conducted, so as it stands this manuscript is not reproducible. For example:

Thank you for pointing this out. We apologize for the limited detail provided initially. We have worked to fix this by presenting full methods for the study, including the additional experiments carried out during this revision.

It is not clear to be how the vesicle analysis was done. Was it from a 2D image, or z-stack? How were vesicles selected to ensure consistent results (in some figures it is hard to differentiate between a large vesicle or two very close together)?

We generated Z-stack image series of BICs in the Zeiss and Leica confocal microscopes and collapsed images series to improve vesicle resolution. However, when vesicles were so close of each other, the distinction between two or more vesicles was carried out by looking at the individual images in the Z-stack. We now provide a full explanation of the new Image J-based methodology used.

How is co-localization calculated? Is it manual, did it use a fiji plugin, what are the parameters and thresholds used?

Co-localization in BICs is straightforward to observe in our leaf sheath preparations of rice blast infections. We did not use a fiji plugin, which did not seem necessary in our case. We did use fluorescence intensity linescans in some cases to show the coincidence of fluorescence intensities in vesicular BICs.

What is the threshold of a swollen/regular BIC?

Cytoplasmic effector fluorescence in swollen BICs is 2.5 - to 3-fold larger than in normal BICs and it is often irregularly-shaped after CME inhibitor treatment, compared with the DMSO control. Distinct vesicles were not observed in BICs after either VIGS or chemical inhibitor treatments.

While I appreciate how difficult it is to study endocytosis in knock down plants, the authors did a great job using many pharmacological agents to complement these findings. I wonder why the plant CME inhibitor ES9-17 was not used - as to date this appears to be the most robust and characterized in plants (Dejonghe et al 2019)?

We are grateful for this suggestion and have now added data using ES9-17 and its precursor inhibitor ES9. The data obtained using these direct inhibitors of clathrin heavy chain are consistent with our observations with VIGS and the other inhibitors used and therefore further support our conclusions.

The figures of the manuscript are currently using red and green, to be more accessible the authors should use a color-blind friendly scheme (like magenta and green).

We are grateful for this suggestion. All images have now been converted to magenta and green and graph colors

chosen to be color-blind friendly.

Minor comments:

Line 36 - "VIGS" should be written in full for clarity

DONE

Line 180 - I am not sure I understand how a novel effecter was identified, so more details would improve this statement.

We have added the information that we previously identified Bas83 as a 36-fold upregulated effector-encoding gene during biotrophic invasion (Mosquera et al Plant Cell 2009). We identified the distinct early localization signal under appressoria and subsequent membrane vesicle localization pattern in this study. We searched for Bas83 homologs in the *M.* oryzae genome and identified Bas170 which had a low level of similarity but a conserved intron. The sequence similarity is shown in new Supplemental Figure S3.

Line 187 - please expand how the vesicles are similar.

Thank you for this comment. We were trying to point out that the fluorescent Bas170 shows a similar localization pattern to previously characterized effectors in both BIC developmental stages, but also highlighting that the localization underneath the appressorium is unique to Bas170. We now point out a recent report of another effector, Mep1 (Yan et al., 2022 BioRxiv. (https://www.biorxiv.org/content/10.1101/2022.07.18.500532v1), which suggests that appressorial pore localization may become a common feature in blast effectors.

The authors often describe results as 'massive colocalization'. It would be better to provide a quantitative value. (eg line 267).

This has been changed.

Line 367 - CEM should be CME

Corrected

Table 1 - Perhaps Dutta & Donaldson 2012 should be referenced here

Thank you. This has been done.

"hpi" is only defined in figure 10.

Now defined at first use in the text.

Fig 8 - the Y axis is not "pixels", should be intensity

Changed

Reviewer #2:

This is an interesting study that seeks to provide a better understanding how effectors of the fungal pathogen Magnaporthe oryzae translocate inside rice cells. This is very challenging work, especially when trying to examine the roles of clathrin-mediated and clathrin independent endocytosis during infection. At his point, the work is based on (co-)localization studies combined with chemical and genetic interference and provides evidence that effectors are translocated via clathrin-mediated endocytosis. This is very exciting work and does contribute substantially to our understanding of plant-fungal interactions. Before this work is published, however, some questions regarding data presentation should be addressed, as detailed below.

Thank you for your support for the importance of our work. We feel that this reviewers' comment also addresses the 'novelty' issue regarding the role of CME in effector delivery raised by Reviewer 1.

Figure 1C, 1F: "Bar charts are based on means of three biological reps (data points shown); error bars indicate standard deviation." Could the authors please clarify whether this is averaging the means of the three replicates or the mean of all data from the three replicates? The first case seems not correct. Please check throughout all figures.

Thank you for pointing out our need for clarification on this point. The bar charts are based on the mean of combined data from all three replicates, and the plotted points show the means of each individual biological rep. We have clarified this in every figure legend where relevant. Where relevant in other figures, we show individual data points for each replicate.

Figure 2D and lines 202ff: "Whereas BICs on primary hyphae deliver effectors in 'micro-vesicles' ranging from 41 nm to 114 nm, BICs on mature bulbous hypha contain effectors in 'macro-vesicles' ranging from 153 nm up to 2310 nm at times". What is the resolution of the used confocal microscope and is it possible to image structures small as 40 nm?

Thank you for pointing out this problem, which was also raised by Reviewer 1. We have re-calculated all vesicle measurements in ImageJ and now report realistic numbers. We furthermore provide detail of the methodology used in calculating the size of vesicles to enable independent repetition of these measurements in future.

Figure 3A: Can the authors explain the patchy appearance of the LTi6B:GFP plasma membrane marker?

The patchy appearance in the invaded plant cell may be an indication that the invaded host cell is not completely healthy. Most of the patchy fluorescence observed is in the vicinity of the appressorium/penetration site, which is consistent with there being some potential effect on the membrane at the point of penetration and invagination of the plasma membrane to form the EIHM.

Lines 218ff: "Here we again use LTi6B:GFP-labeled rice to confirm that BICs contain abundant plant plasma membrane (Figure 3A,B). Individual effector vesicles were observed to be surrounded by labeled plant plasma membrane (Figure 3C), especially for vesicles in side-BICs on mature bulbous hyphae, where the vesicles reach diameters of 150 nm or larger." Lines 239ff: "Taken together, these results indicate that BIC- associated effector vesicles are more likely derived through plant endocytosis than from secretion of fungal exosomes." In order to make this conclusion, additional evidence is needed. FM4-64 labels any lipid compartment including extracellular vesicles. The authors should investigate LTi6B:GFP at the BIC and effector co-localization under conditions of plasmolysis, and use co-localization with TET8 or PEN1 as markers for extracellular vesicles.

We thank the reviewer for pointing out that this section was not clearly written. We have amended the sentence to say "results from staining of rice plasma membrane with both LTi6B:GFP membrane fusion protein and endocytosis tracer dyes are consistent with the formation of BIC-associated effector vesicles through plant endocytosis". We have therefore not mentioned fungal exosomes explicitly.

We previously showed that FM4-64 is excluded from biotrophic invasive hyphal membranes, most notably visible in IH septa and vacuolar membranes, by the brightly stained EIHM enclosing invasive hyphae (Kankanala et al., 2007; Giraldo et al., 2013.) Indeed, in Figure 3D, the fungal septa and vacuolar membranes (there is normally a large vacuole in the BIC-associated cell) in each IH are not stained, indicating that the EIHM is intact around these IH. In an additional FM4-64-stained IH with a side-BIC (Supplemental Movie S2), the stain is again excluded from fungal PM/septa and vacuolar membranes, confirming that an intact EIHM is present and indicating that fungal exosomes would not be stained with FM4-64 without merging with or crossing the EIHM. Likewise, IH/BIC biology is not conducive to learning anything through plasmolysis, since the IH is always included inside the plant protoplast produced by plasmolysis and the EIHM does not move away from the IH during plasmolysis (Kankanala et al., Plant Cell, 2007).

We have modified our concluding sentence so it does not mention fungal exosomes. We agree that it would be interesting in the future to carry out an investigation of fungal exosomes/EVs in *M. oryzae*, but this is clearly beyond the scope of this study. The co-localization experiment to investigate TET8 and PEN1, which are established markers for plant extracellular vesicles, would however simply identify whether such vesicles are present at infection sites, but it would not reveal anything about fungal exosomes. We are not sure how plant EVs could be directly involved in effector translocation and therefore do not understand how this experiment would be informative for our specific goal. However, we agree that defining the role of both fungal exosomes and plant EVs is important to carry out in future.

Figure 5A: Please also show maximal projections to clearly view the cortical actin cytoskeleton.

The goal of our current experiments is not to determine the role of actin and given Reviewer 1's comments of the role of plant actin in endocytosis not being clear, we decided not to focus as much on this aspect of the study. We simply show the localization at BICs.

Figure 7 and lines 301ff: "However, as expected, silencing of these genes caused stunting and decreased plant health (Supplemental Figure S5), and levels of pathogenicity in blast disease are known to be decreased on unhealthy rice plants." The infections were performed 15 days post silencing. Would it be possible to shorten the time post silencing, thereby avoiding effect on plant development?

Rick Nelson, who developed this VIGS assay, recommended we do assays at 15 dpi. Also, in our experience, this is the time when stunting begins to appear, ensuring that widespread silencing is occurring. Performing infection assays earlier may result in silencing not being uniformly activated, with some infected cells not showing silencing. For this reason, we did not carry out the experiments at earlier time points. These experiments are extremely time-consuming and we did our best to undertake them at a time in which we would be best able to ensure silencing had taken place. We have been clear about limitations of our study.

Lines 304ff: "Silencing of either CME component RNAiAP-2 or RNAiCHC1 led to a distinctive swollen BIC phenotype, with enlarged, abnormally-shaped BICs that lack effector vesicles (Figure 7C-F)." This phenotype is very interesting and should be explained. Is exocytosis in infected plant cells upregulated and less balanced with CME because of the genetic interference? Does LTi6B:GFP still found at BIC?

We are grateful to the reviewer and now provide more information on this point. We wouldn't expect silencing of plant CME components to impact secretion of cytoplasmic effectors by the fungus. The swollen BIC phenotype would be expected if cytoplasmic effectors are still being secreted from the fungus, but they accumulate inside the BIC instead of being translocated into the plant cytoplasm. If effector vesicles are being formed through plant endocytosis, there should not be visible vesicles if endocytosis is inhibited and the effectors should be retained inside the EIHM. Indeed, we don't see distinct effector vesicles in the swollen BICs formed by inhibiting CME, either via VIGS or chemical inhibitors. We haven't silenced CME in LTi6B:GFP labeled plants, which are not in a rice background showing effective silencing with the VIGS vectors used. However, localization of cytoplasmic effectors in swollen BICs, and not dispersed throughout the host cell implies the EIHM, which always labels with LTi6B:GFP, is intact.

EM analysis of normal and swollen BICs would provide additional insights.

We agree that providing further EM analysis would be potentially informative. However, we also feel that such analysis is beyond the scope of this submission. Carrying out EM analysis of plant infections is a major undertaking because random sections through infected tissue rarely show BICs, so many hundreds of sections must be generated. The information obtained would also not indicate whether these BICs are still active in translocating effectors, in contrast to live-cell imaging. Therefore, carrying out EM is a significant undertaking that would require correlative light and EM; specifically requiring focused sectioning to identify active BICs regions through fluorescence microscopy. Success also requires sample preparation by high pressure freezing and freeze substitution to preserve membranes. We would like to carry out such analysis in future but recognize this would be a 3-5 year project of focused work to be meaningful.

Figure 8B and Table 1: The authors correctly list Cantharidin and Triclosan as PP2C inhibitors, affecting flg22-induced FLS2 endocytosis. However, being PP2C inhibitors, they target signaling components and are not prototypic CME inhibitors. Please make respective text changes, and instead explore Endosidin 9 as inhibitor of endocytosis.

We are grateful for the suggestion. Endosidin 9, and the less toxic derivative, Endosidin9-17 (ES9-17), were published in 2019, after we completed the chemical inhibitor portion of this project. We have now added the new data based on using these more specific inhibitors. Experiments with both CHC-specific inhibitors, including ES9-17 with fewer side effects, support our conclusions.

Minor point:

Line 366ff: "We report that BICs producing effector vesicles contain multiple hallmarks of active clathrin-mediated endocytosis (CEM),..." should be CME.

CHANGED

<u>Reviewer #3</u>:

The manuscript "Clathrin-mediated Endocytosis Facilitates Internalization of Magnaporthe oryzae Effectors into Rice Cells" by Oliveira-Garcia et al showed that clathrin may play a critical role in facilitating the internalization of fungal effectors that are targeted to the interior of host cells. A host cell component, Clathrin component, and actin were all found localized at top-BIC. Knockdown of clathrin component expression or addition of clathrin inhibitors affected the development of BIC and fungal infection. Several transgenic rice lines expressing different organelle markers were used, making the co-localization results are convincing.

Thank you for agreeing that our co-localization results are convincing. We also believe this comment addresses the 'novelty' comment of Reviewer 1, as this reviewer clearly sees the importance of our study.

While the results suggested that clathrin may be critical at tip-BIC, I am not convinced that clathrin-coated vesicles (CCV) are generated or the vesicles shown in Figures 1-3.

A. The sizes of putative vesicles are quite diverse (40 - 2310 nm) and very different from the relatively uniform sizes of CCV observed in plants and other organisms.

We have remeasured effector vesicle sizes, which range from ~200nm to more than 1000 nm. However, the reviewer is correct that the smallest vesicles are still significantly larger than traditional CCVs, and that vesicles become larger and less abundant as the fungus grows inside a host cell. We report the sizes that we observe and provide details of

the methods to allow others to independently carry out such measurements. We do not know the reason for the difference in size observed. However, there is no reason to assume that fungal-hijacked plant endocytosis would necessarily produce exactly the same-sized CCVs. Clearly, the vesicles increase in size as IH differentiate and grow to fill host cells. Some fungal induced factor may therefore be influencing the size of effector-containing vesicles. Perhaps the effector vesicles are fusing and less effective at releasing their contents as host cell invasion progresses. We accept that this will need further investigation and we have been very measured in our comments and interpretations.

B. Based on the model, CLC1-eGFP should also be detected at the vesicles but no vesicles were shown in Fig. 6A. Why CLC1-eGFP was not detected at vesicles? One would expect the signal is similar to that of Bsa83.

Thank you for this comment because we have now repeated these experiments and re-analyzed all of our previous data. We have replaced the original images in Figure 6A with new representative images that show individual CLC1:eGFP vesicles in tip- and side-BICs. We have also added a new panel, Figure 6B, showing a side-BIC with individual clathrin-coated vesicles and we highlight the correspondence between the CLC-1 and Pwl2-effector fluorescence signals with fluorescence intensity linescans. This image is one z-section from Supplementary Movie S3, which was included in our initial submission. Additionally, we have added new data with additional cytoplasmic effectors, showing Bas1- and Bas170-labeled vesicles co-localizing with CLC1:eGFP (Sup. Fig. S6).

The fact that both signals of OsCLC1-eGFP and LifeAct-eGFP were very strong and colocalized well with Pwl2-mRFP at tip-BIC may suggest a bleed through of the strong signal of Pwl2-mRFP.

We have extensive experience with cytoplasmic effector:mRFP fluorescence and have shown we are not getting bleed through in the GFP channels. This was essential to previous studies from the Valent group (Kankanala et al., 2007 Plant Cell, Mosquera et al., 2009, Plant Cell, Khang et al., 2010 Plant Cell), as well as collaborative work from the Talbot and Valent groups (Giraldo et al., 2013 Nat. Comms; Sakulkoo et al., 2018 Science). We always check for potential bleed through of fluorescence signals. An advantage of our collaboration is that we can independently verify co-localizations in both laboratories to validate results. The new Figure 6A shows a tip-BIC showing CLC1:eGFP vesicles colocalizing with Pwl2:mRFP and some BIC-associated vesicles containing only CLC1:eGFP (arrowhead in the BIC at 19 hpi) that rules out the hypothesis of bleed through of the strong signal of Pwl2-mRFP. In addition, the parallel work that we did with the CIE marker flotillin serves as an internal negative control for bleed-through, since the same Pwl2:mRFP transformant was also used in these experiments and the majority of infection sites lacked co-localization with flotillin1:GFP.

It is not clear how Bsa83 is related to Bwl2 internalization and the purpose of including Bsa83 in the manuscript.

Thank you for pointing out that we did not explain this as well as we should have. Our two new effectors, Bas83 and Bas170, both naturally accumulate under appressorial pores, indicating that these effectors are secreted very early, and this phenotype resembles the phenotype from Pwl2, that is accumulating under appressoria after inhibition of CME. Interestingly, Bas83 appears to label empty membrane vesicles focused around the BIC-associated fungal hyphae, and it is a reasonable hypothesis that this effector might be involved in recruiting and replenishing plant membrane that is depleted through extensive endocytosis at the BICs. We tried very hard to generate a targeted deletion mutant of Bas83, but we did not succeed in either of our laboratories. It is still most likely an important determinant of biotrophic invasion of rice, and may also contribute to viability or membrane dynamics.

The conclusion heavily relied on confocal microscopy. To make the results more convincing, immunogold labeling assay should be employed for the colocalization of clathrin and Pwl2 in the vesicles.

As mentioned previously, we agree that EM analysis would informative in future. However, this is a major undertaking because random sections through infected tissue rarely show BICs and also do not indicate if these BICs are still active. Carrying out immuno-localization is a significant undertaking, requiring correlative light and EM; specifically requiring focused sectioning of active BICs regions identified through fluorescence microscopy. In addition, developing immune-gold EM methods in conjunction with high pressure freezing/freeze substitution of infected plant material is very challenging. This data will require focused work in the future and a considerable amount of time to complete properly. We believe that this is beyond the scope of this study.

TPC2022-RA-00359 1st Editorial decision – *revision requested* **Jan. 20, 2023**

First, we apologize for the length of time that your manuscript was under consideration; one of our reviewers was delayed in responding, and the holiday break may have played a role.

We have received reviews of your manuscript entitled "Clathrin-mediated Endocytosis Facilitates Internalization of Magnaporthe oryzae Effectors into Rice Cells." Thank you for submitting your best work to The Plant Cell.

Although this work has generated some controversy among reviewers, the editorial board agrees that the work you describe is substantive, falls within the scope of the journal, and may become acceptable for publication, pending revision and potential re-review. We ask you to pay particular attention to addressing concerns and questions raised by the 4th Reviewer in preparing your revision. However I should stress that we are reluctant to see manuscripts undergoing multiple rounds of revision and would be unlikely to offer you more than one chance to satisfy the reviewers. Please contact us if there are ambiguous comments or if you wish to discuss the revision.

--- Reviewer comments:

[Provided below along with author responses]

TPC2022-RA-00359R1 1st Revision received Feb. 23, 2023

Reviewer comments on previous submission and **author responses**:

Reviewer #2

The authors have sufficiently addressed my concerns and I believe the manuscript is now suitable for publication.

Response: Thank you for the comments that have improved our manuscript.

Reviewer #3:

Authors have substantially improved the quality of results, provided more convincing evidence, addressed my points nicely.

Response: Thank you for the comments that have improved our manuscript.

Reviewer #4:

The manuscript of Oliveira-Garcia et al. brings a set of interesting, mostly confocal microscopy data on the mechanism of how fungal effectors enter rice cells. Clathrin- mediated endocytosis is proposed here to be the major mechanism involved in this process. Besides analyzing previously identified effectors, one new cytoplasmic effector is described here (Bas170), a homolog of the previously described effector Bas83.

We actually show novel localization patterns for both Bas170 and Bas83 effectors in the manuscript. Bas83 was previously identified as upregulated during biotrophic invasion in our 2009 Plant Cell paper, but we report the first localization studies for this effector in this manuscript. We show that Bas83 has an interesting and unique pattern of labeling membrane structures focused around BICs and BIC-associated hyphal cells. We tried very hard to generate a targeted deletion mutant of the *BAS83* gene, but failed in both the Valent and Talbot labs. Dr. Oliveira-Garcia is continuing to work on both of these interesting effectors.

Both of these effectors change their localizations after the application of inhibitors of CMEs. Virus-induced gene silencing was used to decrease the expression of proteins involved in the endocytosis, which blocked the fungus infection. I have some comments that could be taken into account before finishing this manuscript for its publication.

Major comments:

1) I do not think that there exists anything like a specific inhibitor of CIE. The problem is in the definition of CIE. This naming only informs that it is the endocytosis not dependent on clathrin and adaptor proteins. I am happy that authors tested such an impressive set of inhibitors. I got from it that clearly CME is probably involved, but a fluid phase, or microdomain type or sterol- based probably not.

Thank you for this positive comment supporting the major conclusions of this study. We are happy that you agree that our extensive work with a range of inhibitors is consistent with a role for CME in effector translocation. We agree that there is no specific marker for CIE and that most inhibitors have off-target effects. This is precisely why we tested so many inhibitors, in order to identify whether there was a general trend. The analysis provided evidence for the involvement of CME.

2) The choice of FLOT1 protein is not very fortunate because, to be fair, the community of cell biologists does not understand well the function of this protein and its homologs. Yes, I agree that it might be somehow involved in the

microdomain-based endocytosis as some time ago the lab of Prof. Lin showed, but it cannot be used as a marker of this „type of endocytosis" (if it even exists).

Thank you for this comment. We agree that CIE is a not a single process involving one set of components, and that CIE is not well understood in plants. We have edited our manuscript to provide greater clarity with regard to this issue. We chose to work with Flotillin1 based on the report from Dr. Lin's lab (Li et al, The Plant Cell, 2012). However, your point is well taken that Flot1 is not a specific marker for all CIE. We have therefore removed the word/concept of 'endocytosis marker' from our manuscript and modified our writing to better reflect the uncertainties of CIE in plants. In the sections using Flot1, we refer to "Flot1-associated CIE." The chemical inhibitors we used, filipin (sterol binding) and methyl-ß-cyclodextrin (sterol depleting), are generally discussed as targeting CIE, although there is some indication that these inhibitors also have some impact on CME. We had previously acknowledged this issue in Table 1 and discussed the fact that the minor phenotype impact seen with CIE-associated treatments could indicate a minor impact from CIE, or a crossover impact on CME. We have now tried to make all of this clear in the manuscript in response to the points raised.

3) The manuscript would be much clearer if simply authors would concentrate on CME and show in a much better and condensed way that it is the mechanism for the uptake of fungal effectors. This message is very strong.

We view the parallel work with CIE component Flot1, supported by lipid raft inhibitors filipin and methyl-ß-cyclodextrin, as important control experiments. These experiments demonstrate specificity in BIC localization for some endocytosis components, and that the distinctive "swollen BIC" phenotype is specific for CME and not for every treatment associated with plant endocytosis. We have edited the manuscript to downplay conclusions from the CIE-associated experiments (for example, removing CIE from the Figure 10 model figure and legend), and to simply state where our observations are consistent with the involvement of CME. However, we strongly argue to retain the Flot1 experiments in the manuscript. We have worked on better and condensed explanations of our results supporting a role for CME in effector translocation, but these results do provide additional evidence which is consistent with the major conclusion of the paper.

4) On the microscopy level, I think that the only convincing evidence proving the involvement of endocytosis might be provided by using effectors tagged with photoconvertible fluorescent proteins. This approach was already used in plant cell biology several times in past. Simply to photo-convert it in the fungus and then follow it in planta. The authors do not possibly have these proteins ready, so why not to photobleach effectors in fungus and then show that they do not appear as fluorescent molecules in the plant cell, this would be very coinvincing.

We do not currently have photo-activable (PA) fluorescent protein constructs for these effectors. We believe that carrying out such experiments would therefore be beyond the scope of this paper. The photo-conversion of PA-GFP constructs in fungal cells within living plant tissue is also likely to be technically demanding and would require focusing the laser on a tiny area (the BIC/BIC-associated cell) so that translocated effectors would not also be photo-activated after uptake. We have already published the photobleaching experiments that the reviewer suggests, however. We photobleached effectors in FRAP experiments in two previous publications to show that they are continuously secreted into tip-BICs and early side-BICs, even in the presence of BFA that blocks secretion of apoplastic effectors (Khang et al., the Plant Cell, 2010; Giraldo et al, Nature Communications, 2013). We have shown multiple times that BICs regain fluorescent effectors within 3 hours after photobleaching. One of the features of the rice blast system is that we see fluorescent effectors that have been translocated into the cytoplasm and nuclei of invaded plant cells, and also moving ahead into neighboring cells (Khang et al., the Plant Cell, 2010). Therefore, showing that photobleached effectors fail to appear in the host cell would be complicated, because there is a background of effector fluorescence already in the plant cell from ongoing effector translocation. We realize that we did not do a good job of highlighting our data that shows that effector-containing vesicles co- occur with active effector translocation. We have gone back and clarified this aspect of our work by pointing out when cytoplasmic effector fluorescence is visible in the plant cytoplasm in images included in our figures.

5) Line 567 - authors state that „high resolution confocal microscopy images of BICs were generated". I wonder, how is this high resolution defined? Authors state that they used CLSM, the highest with 63x objective, which could hardly give better than 300 nm axial and 500-600 nm in z. In another words, any image analysis of these images could not resolve better than this. No deconvolution is mentioned that is why I am very confused.

Thank you. We agree that 'high resolution' was not the appropriate term to use here. We meant that 'high quality' confocal microscopy images were generated for analysis by ImageJ, meaning images were the highest quality possible under our experimental conditions. We have removed this confusing reference to high resolution to avoid confusion.

6) I disagree that in plant cell biology, we could talk about well-established „markers" for CME and CIE. CLC1 „marker line" cannot be taken as marking the endocytosis, not at all. CLC1- GFP is distributed almost everywhere in the endosomal space, including trafficking pathways for the secretion, vacuolar pathway, recycling pathway, and endocytosis. However,

endocytosis is only a small subset of these, where FLOT1 might be followed. In this work, this pool is not studied at all; for this, one would need at least TIRF-based or HILO-based imaging. Similarly, FLOT1 is kind of everywhere, and again small pool of it would be on the PM (for FLOT1 see my comment 1).

We agree that FLOT1 is implicated in only of subset of potential CIE processes in plants. Clathrin light chain protein is a defining component of CME, together with the clathrin heavy chain, so in that sense it is, however, a well-established marker for CME. However, we have removed all references to "endocytosis markers" from the manuscript. A major point of our clathrin light chain-1 fluorescence experiments was to determine whether BIC regions are enriched in CLC-1 and indeed we observed that the CLC1:eGFP pattern closely follows the cytoplasmic effector fluorescence, consistent with labeling of individual effector-containing membranous compartments. We did not follow the overall distribution of CLC-1, other than to notice that the overall CLC-1 fluorescence pattern in our uninfected cells is consistent with patterns reported in previous studies. Following pools for endocytosis components in other parts of the cell was not relevant to our current study. TIRF imaging is not useful in studying BICs at the fungal-plant interface, because they are found in the interior of the cell, out of reach for this imaging technique. As mentioned in response to previous reviews, we plan to move to electron microscopy for enhanced resolutions, but that will be a major undertaking and is well beyond the scope of the present study.

7) In this manuscript, the word „vesicle" is not used correctly at all. Please refer to textbooks on the definition of the word „vesicle". And there is not even one single vesicle visible and detected in this work, because the microscopy used here simply does not allow to see individual vesicles. They are much much smaller. Everything that is analyzed in this work might be called as endosomes (probably some of them are recycling endosomes), or aggregation of endomembranes. The authors use the following argumentation in their rebuttal: „We do not know the reason for the difference in size observed. However, there is no reason to assume that fungal-hijacked plant endocytosis would necessarily produce exactly the same-sized CCVs. Clearly, the vesicles increase in size as IH differentiate and grow to fill host cells. Some fungal- induced factor may therefore be influencing the size of effector-containing vesicles. Perhaps the effector vesicles are fusing and less effective at releasing their contents as host cell invasion progresses. We accept that this will need further investigation and we have been very measured in our comments and interpretations." This explanation cannot be accepted. All analyzed aggregations of signal might be of endocytic origin, it looks like a probable scenario (based on what authors show here), but they are undoubtedly not vesicles. The best would be to wipe out the word „vesicle" from this manuscript completely. I would even insist on it.

These comments imply that the reviewer agrees with that our findings support internalization of effectors by endocytosis. However, there seems to be confusion on the definition of the term "vesicle'. In our understanding, a vesicle is defined as a compartment formed by a lipid bilayer separating its contents from the cytoplasm or a fluid-based extracellular environment. Another definition is a 'fluid-filled sac' and vacuoles are listed in some places as examples of 'vesicles'. Another is 'a structure within or outside a cell, consisting of liquid or cytoplasm enclosed by a lipid bilayer'. Standard textbook definitions do not indicate a size limit to what can be defined as a vesicle. Endosomes are a very specific type of vesicle in the trafficking pathway, and we do not know whether our effector vesicles are classical endosomes. For this reason, we originally chose the term 'vesicle' and used it according to the very general definition (e.g. Albert's *Molecular Biology of the Cell* glossary "Vesicle: Small, membrane-bounded, spherical organelle in the cytoplasm of a eukaryotic cell". Indeed, we generally referred to 'effector vesicles' to differentiate what we observe from normal CCVs.

We recognize, however, that the reviewer is referring to the definition of 'vesicle' in the context of the plant intracellular transport literature, which defines intracellular transport vesicles as approximately 30 nm to 100 nm in diameter and vesicles within a clathrin cage as typically 40 nm to 100 nm in diameter (Ratamero and Royle, 2019 *BioRxiv* - https://www.biorxiv.org/content/10.1101/555813v1.full.pdf). To avoid confusion with this much more specific definition, and because we do not yet know the actual identity of the structures we observe, we have now adopted the term 'membranous effector compartments'. This simply describes the fact that we observe membrane-bound structures to which effectors localize. We have adopted this term throughout the manuscript and used the abbreviation MEC. We have done this in response to the reviewer comment, as we do not want to use a term that is inconsistent with terminology used by the wider plant cell biology community. We hope this new term addresses the concern.

8) If everything is as it is presented here, one should see endocytosed effectors much earlier after the infection and perhaps, with a super-resolution approach, individual vesicles containing effectors might be seen, perhaps even co-localizing with CLC. In the present form, the manuscript is just showing very late stages after the infection and, yes, I agree, it is quite convincing in showing that they are probably at the very beginning entering plant cells by CME.

We are grateful to the reviewer again for saying it is quite convincing that "effectors" are "probably at the very beginning entering plant cells by CME." These comments point out that we have still not been clear enough in describing BICs and effector biology in the rice blast system. We are not working with "very late stages after the infection". The blast fungus requires around 18 to 20 hours post-inoculation to form appressoria and penetrate into the

host cell. Our data covers 18 to 28 hpi, which includes the period of appressorial penetration, growth of primary hyphae with tip-BICs, differentiation of first bulbous hyphae with side-BICs and the beginning of growth of the non BIC-associated bulbous hyphal cells that eventually fill the initially colonized epidermal cell. Several of our figures show that effector translocation (specifically, significant effector fluorescence in the cytoplasm of the invaded cell, and even in surrounding host cells) is occurring in host cells with BICs and effector vesicles. We have tried to clarify this critical pathosystem biology in the manuscript.

9) Lines 566-576 - I tried my best to understand how „Size measurements of vesicles carrying effector and clathrin fluorescence" were performed, but I totally failed. I only know that it cannot be so as it is mentioned in the rebuttal, i.e. that authors "remeasured all vesicle sizes using ImageJ instead of our microscope software".

The ImageJ Length Measurement Tool is a community standard for making measurements based on information available in digital images. Specifically, vesicle diameters were determined in pixels and converted to nm based on the pixel numbers in the 1000nm scale bars in our images. We have tried to make this as clear as possible in the methods section.

10) Figure legends are, in reality, rather kind of text of results itself. They are extremely difficult to follow, please excuse me, if I am not „in". I would strongly suggest to follow Plant Cell instructions:

- Provide a short, descriptive title.
- Describe each panel; include basic information necessary to understand the figure without referring to main text or methods section.
- Define symbols and abbreviations.
- Define error bars.

Here is the paper from TPC, which has legends much better to follow, if you consider my comment relevant, please confer to https://doi.org/10.1093/plcell/koac014

Thank you for pointing out that our figure legends could be improved, including better short descriptive titles in some cases. We have worked hard to simplify and clarify the figure legends, which are now consistent with TPC guidelines and the example provided.

11) Lines 1023, 1027, 1075, 1088, 1118 - figure captions cannot contain references to other, even supplemental images or videos, in any case. These should be placed consistently in the text. I also found references to other main images in the figure legends, these should be also removed.

We were referring to supporting supplemental figures, or pointing out consistency between images in different figures to help reviewers understand the depth and strength of our data. We have removed these cross references in the figure legends in the manuscript. We understood that we should be clear which Figures/data are supported by supplemental Figures and Movies, and we have left this information in the Supplemental Figures.

Minor comments:

Figure 6 - BICs uniformly contain clathrin, but not in flotillin1 - probably wrongly entitled, right? It cannot be "in flotillin1"

Thank you for pointing out this typo.

1047 - not „magneta", but magenta

Thank you for pointing out this typo.

TPC2022-RA-00359R1 2nd Editorial decision – *acceptance pending* Mar. 2, 2023

We are pleased to inform you that your paper entitled "Clathrin-mediated Endocytosis Facilitates Internalization of Magnaporthe oryzae Effectors into Rice Cells" has been accepted for publication in The Plant Cell, pending a final editorial review by a science editor. The conceptual advance of this research has been well-recognized by both editors and reviewers, and the additional reviewer's comments are reassuring and confirmed our decision. As a result, we have recommended your paper be highlighted with an In Brief due to its high value to both the immunity and cell biology field.

Final acceptance from Science Editor Mar. 20, 2023
